# Compliant lower limb exoskeletons: a comprehensive review on mechanical design principles

**DOI:** 10.1186/s12984-019-0517-9

**Published:** 2019-05-09

**Authors:** Maria del Carmen Sanchez-Villamañan, Jose Gonzalez-Vargas, Diego Torricelli, Juan C. Moreno, Jose L. Pons

**Affiliations:** Neural Rehabilitation Group, Cajal Institute, Spanish National Research Council (CSIC), Avda Doctor Arce, 37, E-28002 Madrid, Spain

**Keywords:** Assistance, Compliant actuation, Mechanical compliance, Mechanical design, Lower limb exoskeleton, Rehabilitation

## Abstract

Exoskeleton technology has made significant advances during the last decade, resulting in a considerable variety of solutions for gait assistance and rehabilitation. The mechanical design of these devices is a crucial aspect that affects the efficiency and effectiveness of their interaction with the user. Recent developments have pointed towards compliant mechanisms and structures, due to their promising potential in terms of adaptability, safety, efficiency, and comfort. However, there still remain challenges to be solved before compliant lower limb exoskeletons can be deployed in real scenarios. In this review, we analysed 52 lower limb wearable exoskeletons, focusing on three main aspects of compliance: actuation, structure, and interface attachment components. We highlighted the drawbacks and advantages of the different solutions, and suggested a number of promising research lines. We also created and made available a set of data sheets that contain the technical characteristics of the reviewed devices, with the aim of providing researchers and end-users with an updated overview on the existing solutions.

**Electronic supplementary material:**

The online version of this article (10.1186/s12984-019-0517-9) contains supplementary material, which is available to authorized users.

## Background

Robotic wearable exoskeletons^1^ have potential impact in several application domains, like industry [1], space [2] and healthcare [3]. In the healthcare sector, this technology is expected to contribute by reducing the clinical costs associated with the assistance and rehabilitation of people with neurological and age-related disorders [3–6]. Research in this area is clearly shifting toward the inclusion of compliant elements (i.e. actuators, structure^2^, etc.) as a way to overcome the main drawbacks of rigid exoskeletons, in terms of adaptability, comfort, safety and efficiency [7].

Currently, there is a large variety of designs of lower limb compliant exoskeletons aimed at gait rehabilitation or assistance. However, there is a lack of detailed information about the mechanical components of these devices, which has been largely overlooked by previous reviews (e.g. [7–9]). These variety and lack of information makes it difficult for developers to identify which design choices are most important for a specific application, user’s need or pathology. For this reason, we aimed to bring together available literature into a comprehensive review focused on existing lower limb wearable exoskeletons that contain compliant elements in their design.

In this work, we refer to ‘compliant exoskeleton’ as a system that includes compliant properties derived from non-rigid actuation system and/or structure. Our review focused on three particular aspects: the actuation technology, the structure of the exoskeleton and the interface attachment components^3^.

We have gathered the mechanical and actuation characteristics of 52 devices into standardized data sheets (available at Additional file 1), to facilitate the process of comparison of the different solutions under a unified and homogeneous perspective. We consider that such a comprehensive summary will be vital to researchers and developers in search for an updated design reference.

### Methodology

We applied the following search query on the Scopus database: TITLE-ABS-KEY(“actuat*” AND (“complian*” OR “elastic*” OR “soft”) AND (“exoskeleton*” OR “rehabilitat*” OR “orthotic*” OR “orthos*” OR (“wearable” AND “robot*”)) OR “exosuit” OR “exo-suit”), which returned 1131 studies. We excluded: publications focusing on upper limb robots; non-actuated compliant exoskeletons; solutions where compliance was achieved through control; studies that did not report any mechanical information on the robot; and studies not related to either assistance or rehabilitation. The above process resulted in a total of 105 publications, which covered 52 different lower limb exoskeletons.

To simplify and structure the information, we classified the compliant exoskeletons according to the mechanical component that results in their intrinsic compliant performance: (i) exoskeletons with compliant actuators (i.e. series elastic, variable stiffness and pneumatic actuators) and rigid structure; (ii) exoskeletons with soft structure (soft exoskeletons^4^) and rigid actuators; (iii) exoskeletons with compliant actuators and soft structure. The review describes the different design choices of the exoskeletons, i.e. actuation system, structure and interfacing attachment components to connect the actuators with the human body.

A glossary with the most commonly used terms in this article has been added at the end of the document. Some definitions have been readapted from the literature.

## Results

**Fig. 1:**
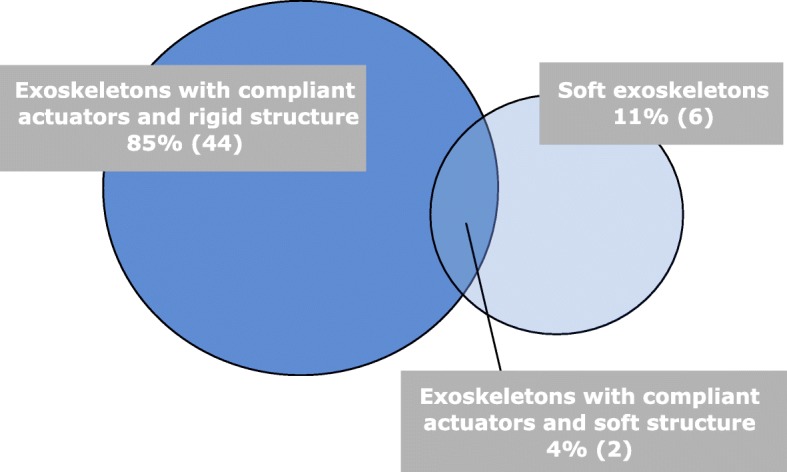
Classification of the 52 lower limb exoskeletons according to their compliant mechanical component

As shown in Fig. 1, 85% of the reviewed articles (corresponding to 44 exoskeletons) used compliant actuators and a rigid structure. Soft exoskeletons represent 11% of the reviewed articles (6 exoskeletons). Two exoskeletons (4%) belong to the intersection of previous groups, this is, exoskeletons integrating both soft structure and compliant actuation^5^. We refer to the latter as “fully compliant exoskeletons”.

### Exoskeletons with compliant actuation

#### Actuation

**Fig. 2:**
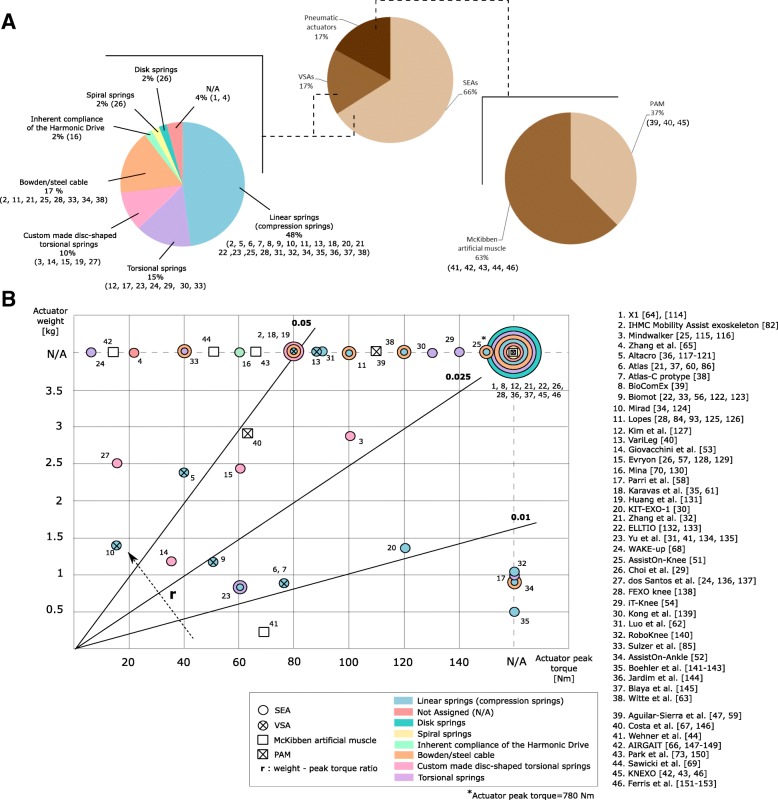
Charactesristics of the studied exoskeletons with compliant actuation^11^. **a** The exoskeletons are classified according to their compliant actuator. Exoskeletons with SEAs and VSAs are classified according to the elastic component type. Exoskeletons with pneumatic actuation are classified according to the pneumatic artificial muscle type. **b** The graph represents the relation between exoskeletons weights and maximum delivered torques. The elastic component and the pneumatic artificial muscle types are also shown

In this group we found three types of actuations systems: Series Elastic Actuators (SEAs), Variable Stiffness Actuators (VSAs), and pneumatic actuators. As shown in Fig. 2-a, 31 exoskeletons use SEAs, which makes this actuation the most popular choice. SEAs are characterized by having an elastic element with fixed stiffness placed in series with the motor or the motor train, and before the actuator load [10, 11]. The use of SEAs has shown improved performance in terms of human-robot interaction, safety, energy efficiency, shock tolerance and backdrivability^6^, when compared to stiff actuators [8, 12–15]. The deformation of the elastic component can also be used to measure the joint torque, thus reducing the need of force sensors [16]. In addition, in spite of their reduced bandwidth [17], they demonstrated better torque tracking during walking in exoskeleton experiments [18].

Variable Stiffness Actuators (VSAs) are implemented in eight exoskeletons. These actuators are a variation of SEAs, in which the degree of compliance can be mechanically modulated to change the actuator’s output characteristics (e.g. output stiffness) [19]. These actuators have the theoretical ability to reproduce the human-like joint stiffness profiles, adapt to environmental changes, and reduce energy expenditure [20, 21].

Figure 2-A shows a classification of these actuation solutions based on the type of elastic element. Most actuators (23) use linear springs, due to commercial availability, ease of implementation and low cost. In spite of their very approximate linear deformation characteristic, these springs present hysteresis [22], which should be compensated to reach fine control [23]. Dos Santos et al. [24] suggested that connecting the load of the actuator in a direct-drive configuration can reduce the hysteresis and residual deflection. Torsional springs are implemented in seven exoskeletons. Five exoskeletons use springs based on a monolithic disc-shaped design. These springs are compact, lightweight, able to withstand high torques with low intrinsic stiffness and are usually custom-developed [25]. There is a wide variety of manufacturing materials, such as maraging steel (martensitic steel with aging treatment) [26] or high-grade titanium [25]. The geometry of monolithic disc-shaped springs is usually defined through an iterative Finite Elements Analysis (FEA) simulation-process [27]. This process has to be carried out carefully to make sure that the spring is able to withstand the expected deformations [16]. However, results from simulations often do not match experimental results, for instance with respect to the actual stiffness [16, 26]. Bowden cables, in combination with linear springs, are used in eight of the reviewed works. These cables allow the motor to be placed away from the actuated joint [28]. The main drawback of this solution is friction, which can be managed through control [28]. Spiral springs are used in one device [29].

Figure 2-B shows the relationship between the peak torque and the weight of the actuators, classified by type of actuator. We observed that this ratio is not proportional. For instance, the actuator presented by Beil et al. in [30] delivers 120 Nm and weights 1.38 kg while the actuator presented by Wang et al. in [25] delivers less torque (100 Nm) and weights 2.90 kg. The weight values of the actuators include data related to the mechanical components (i.e. motors, springs, pneumatic muscles, transmissions, etc.) without considering power supplies or wiring.

The selection of the spring stiffness is critical when designing a SEA or a VSA. Multiple selection criteria have been used [18]. The most common criterion is to set the spring stiffness as the slope of the desired torque-angle profile [25]. Another common principle is based on maximising the energy stored and released throughout the gait cycle [31, 32]. Stiffness has also high implications on control. High stiffness increases impedance, whereas low stiffness decreases bandwidth [10]. In VSAs, stiffness can be changed manually, e.g. through a screw [33–35], or with motors [36–40], through either pretension of the elastic element or a lever arm mechanism with a variable position pivot.

**Fig. 3:**
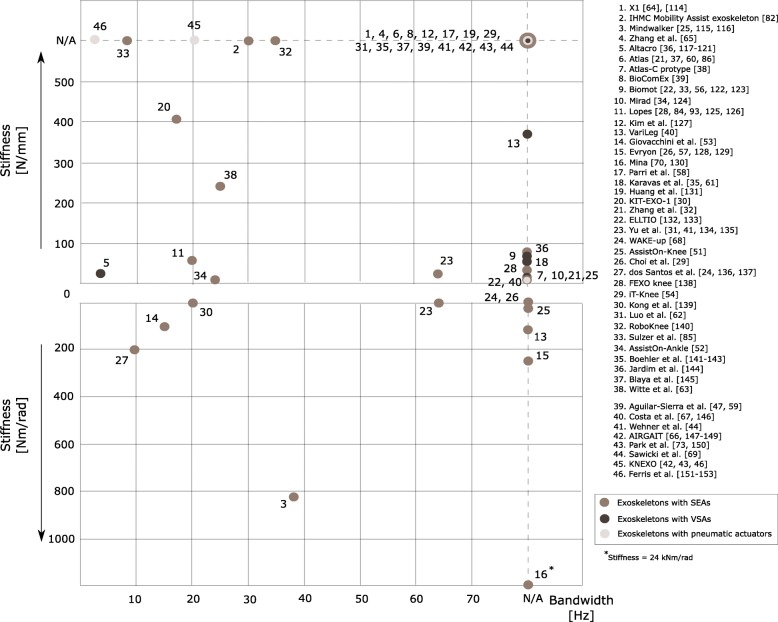
Relation between actuation maximum bandwidth and spring stiffness^11^

Figure 3 compares the spring stiffness values with the resulting actuation bandwidth. The lack of information related to the actuator bandwidth is apparent. The actuators of the KNEXO exoskeleton and the exoskeleton developed by Yu et al. [41] present the highest bandwidth among all compliant exoskeletons [41, 42], as shown in Fig. 3.

Compliant or spring-like behaviour can also be achieved by pneumatic actuators, which depend on input air flow rate to contract and/or expand [19]. Eight of the reviewed exoskeletons use this actuation solution. In contrast to SEAs, pneumatic actuators generate forces through compressed air [43]. These actuators, also known as Pneumatic Artificial Muscles (PAMs), stand out because of their low weight (without considering the compressor for air supply), backdrivability, cost and the high specific force and power they can exert [44, 45]. Among them, the McKibben-type pneumatic muscles [45] are implemented in five of the reviewed exoskeletons (see Fig. 2-A). These actuators consist of an expandable rubber tube surrounded by a textile mesh for tension transmission. Antagonistic configuration of pneumatic muscles can be also used to obtain bidirectional rotational actuation [42]. This configuration also allows to change the effective compliance that is applied to the joint [43]. The main limitations of PAMs are the high hysteresis [46] and nonlinear force-contraction characteristics [43]. This results in complex mechanical design and control [47], particularly when large ranges of motion and high torques are required. Special controllers (i.e. torque controllers) have been proposed to deal with these non-linearities [42, 43]. A particular type of PAMs, i.e. the Pleated Pneumatic Muscles (PPAMs), showed reduced hysteresis [42, 46].

#### Structure

Exoskeletons with compliant actuators normally have rigid structures, composed of mechanical links and transmission mechanisms placed in parallel with the user’s limbs. This rigid configuration hinders a full kinematic compatibility with human joints [48, 49]. In order to cope with this issue, Cempini et al. [50] proposed an analytical method based on a kineostatic analysis of the coupled mechanism of the robot and the human. Other exoskeletons use mechanisms to self-align the axes and reduce the time to dress the exoskeleton on. For example, Celebi et al. [51], implemented a Schmidt coupling actuated by a SEA in order to improve ergonomics and comfort. The AssistOn-Ankle exoskeleton [52] includes a self-aligning parallel mechanism, whereas the exoskeleton presented by Giovacchini et al. [53] has slots in order to modify the structure length and guarantee the alignment. Also, Saccares et al. [54] included five passive Degrees of Freedom (DoF) in a knee exoskeleton to automatically adapt itself to different users. The solution proposed by Junius et al. [55] improved joint alignment by using 3 passive DoF with sliders and hinges.

To adapt to the user-specific morphology, the foot length, pelvis width and inter-joint distance are the primary design parameters. Moltedo et al. [56] proposed a footplate that can be manually adjusted to match different foot sizes and align the ankle joint. Giovacchini et al. [53] presented an exoskeleton whose pelvis structure can be modified in width. Telescopic structures and sliders are most commonly used to adapt to a wide range of user’s height [25, 57–59].

**Fig. 4:**
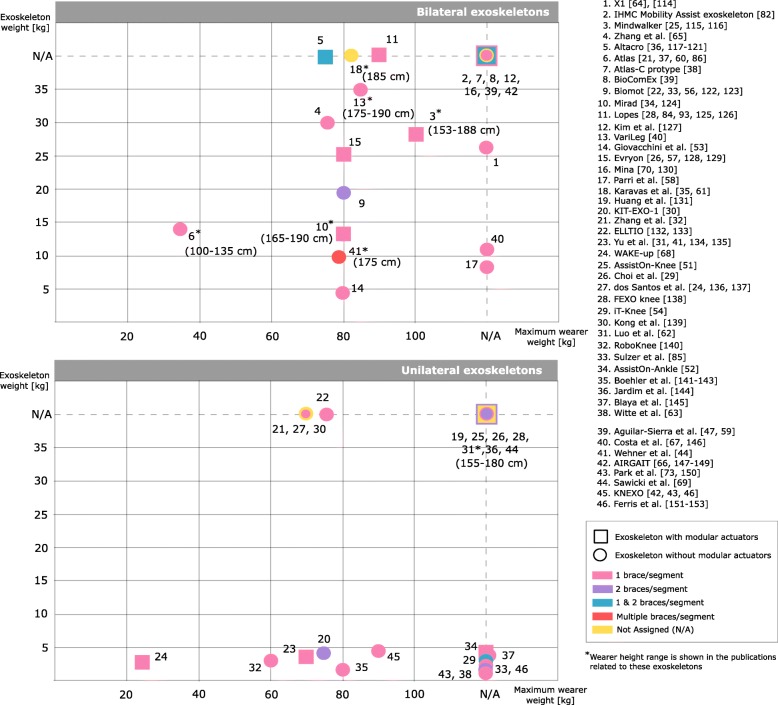
Relation between exoskeletons weights and maximum wearer weights^11^. The graph also shows the number of braces per segment of the robots, its configuration (unilateral/bilateral) and if the exoskeleton has modular actuators

Figure 4 shows the exoskeleton weight as a function of the maximum accepted user’s weight. When available, we also included the height range [25, 34, 40, 44, 60–62]. The average maximum adult wearer weight considered in the reviewed exoskeletons is 100 kg [25], whereas the maximum adult wearer height is 190 cm [34, 40]. Bilateral exoskeletons are heavier than unilateral ones, presenting an average weight of 18.56 kg and 2.52 kg respectively. The weight of bilateral exoskeletons ranges from 4.2 kg [53] to 35 kg [40], whereas unilateral exoskeletons range from 0.87 kg [63] to 4.5 kg [42] in weight. Exoskeleton with series-elastic actuation are heavier than those with pneumatic actuation (see Fig. 4) [25, 26, 40, 64, 65]. It is worth mentioning that the pneumatic actuation usually depends on off-board pressure supplies with a negative impact on portability while favouring lighter structures [42, 59, 66, 67]. Additional details are available in the Additional file 1.

#### Interface attachment components

The braces, cuffs, straps and orthopaedic components used in the reviewed exoskeletons are based on a broad variety of materials and configurations. The majority (30) of exoskeletons with compliant actuators have only one brace per leg segment (i.e. thigh or shank). Five exoskeletons have two braces per segment. Five other exoskeletons present a combined solution, i.e. two braces for thigh and one for shank. Some solutions are based on orthopaedic commercial braces in order to reduce costs [30, 34, 40], but most of them adopted custom-made designs. Rossi et al. [68] present a customized brace made with a 3D printer from a model obtained from a 3D scanner. Moltedo et al. [56] and Sawicki et al. [69] use braces made of carbon fiber. This material ensures power transfer in the sagittal plane of motion while allowing for passive motion in the other two planes. The shape of the braces influences comfort, which is an essential requirement for ergonomics, whereas the fastening mechanism affects the time of dressing^7^ on and off^8^. This process can be simplified with similar solutions such as the double-tier beleaguered structure design of the exoskeleton developed by Zhang et al. [32]. In the solution presented by Neuhaus et al. [70], leg braces were designed with an opening of approximately 180 degrees in order to improve the ease of dressing on and off. Rigid parts of these braces are designed to be attached to the leg where soft tissue’s deformation is minimal (e.g. calf) in order to optimize force transmission between exoskeleton and user’s limbs. The thigh and shank cuffs of the exoskeleton developed by Costa et al. [67] are moulded structures tailored to the user. Position and orientation of the thermoplastic shells of KNEXO exoskeleton are adjustable with a slider mechanisms [42]. Padding covering braces with adaptable positions to user’s preference is sometimes used in order to improve comfort [30].

### Soft exoskeletons

Exoskeletons structures composed by non-rigid components such as textiles take advantage of compliance for providing compatibility with human kinematics and dynamics, with potential to improve comfort, safety, efficiency and functionality [8, 71–73]. Exoskeletons with soft structures are commonly known as soft exoskeletons [74]. We found six robots belonging to this category (see Fig. 1), which represent the 11% of the exoskeletons here reviewed.

#### Actuation

**Fig. 5:**
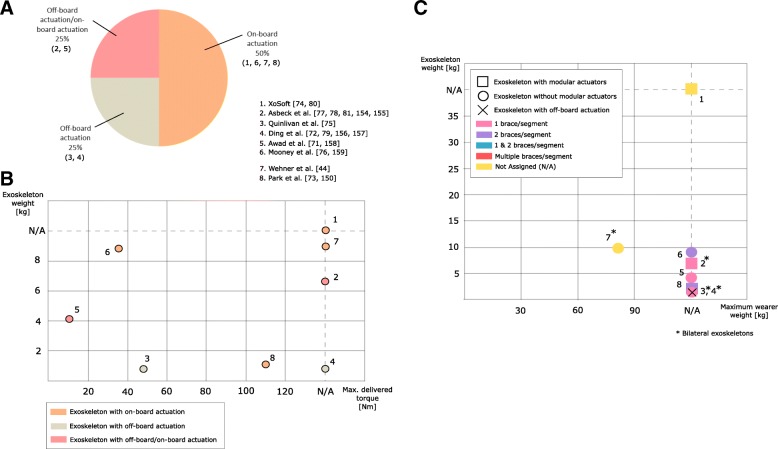
Characteristics of the studied soft exoskeletons^11^. **a** The exoskeletons are classified according to the position of the actuators. **b** The graph represents the relation between exoskeletons weights and maximum delivered torques. The position of the actuators is also shown. **c** Relation between exoskeletons weights and maximum wearer weights. The graph also shows the number of braces per segment of the robots and their configuration (unilateral/bilateral)

The actuation mechanism of soft exoskeletons is usually composed of motors with different gearbox mechanisms (i.e. pulleys [75, 76], gear [72]), which deliver certain torques to the user’s joints though flexible transmissions and textiles worn by the user [77]. Actuators can be placed off-board [72, 75], at the waist level or in a backpack [71, 74, 78], (see Fig. 5-A). The most common flexible transmission used is Bowden cable. Other types of transmission have been proposed, e.g. inextensible cords [76]. The main disadvantages of using cables are slacks and hysteresis [78]. Both can be minimized through appropriate control strategies [79]. A recent approach uses linear compression springs in series with Bowden cables in order to decrease the overall hysteresis [78]. The XoSoft exoskeleton [80] proposed a new type of actuation principle based on custom-made soft clutches.

#### Structure and interface attachment components

The structure of soft exoskeletons is mainly composed of textiles made of neoprene and/or others flexible materials [74]. Velcro-covered tabs have been proposed to improve adaptation of the textiles to the user [78]. As these exoskeletons transmit torques through biological joints by applying tensile forces, they do not constrain wearer’s joints. This minimizes undesirable interferences with gait biomechanics, overcoming in this way the problem of misalignments [76, 81].

We present in Fig. 5-B the relationship between soft exoskeleton weights and delivered torques. Weights of exoskeletons with off-board actuators do not include the actuators weight. Among the on-board solutions, the exoskeleton designed by Mooney et al. [76] delivers 35.6 Nm during ankle plantar flexion with a total weight 8.96 kg, considering power supply and actuators weight. Within the off-board applications, the exoskeleton delivered by Quinlivan et al. [75] has a weight of 0.89 kg and delivers the highest torque value (48.35 Nm).

The average weight of unilateral and bilateral exoskeletons is 4.67 kg and 4.37 kg respectively. The maximum weight is 9.12 kg for bilateral exoskeletons [44] and 8.96 kg for unilateral exoskeletons [76]. The minimum weight is 0.95 kg [73] and 0.86 kg [72] for unilateral and bilateral devices respectively.

The weight of soft exoskeletons with off-board actuators fluctuates within a narrow range, i.e. 0.86–0.89 kg [63, 75], whereas solutions with on-board actuators spans from 0.95 kg [73] to 9.12 kg [44]. The maximum accepted user’s weight and height is not reported in the majority of the publications reviewed.

In soft exoskeletons, the attachment components, such as braces and straps, are part of the textiles and compliant structure. Four soft exoskeletons have one brace per segment and two exoskeletons have two. The XoSoft exoskeleton [74] includes a custom garment designed to fit the user.

### Fully compliant exoskeletons

Two out of the 52 reviewed exoskeletons have both compliant actuation system and structure. The exoskeleton developed by Park et al. [73] integrates four McKibben artificial muscles on the ankle joint and uses textiles and carbon fiber to reinforce foot and shank structures. It can apply up to 110 Nm for ankle dorsiflexion and has a weight of 0.95 kg. The exoskeleton presented by Wehner et al. [44] uses 8 pneumatic actuators and a soft structure with multiple textile straps. Its soft structure was designed considering the virtual anchor concept. They designed the distribution and location of the textiles to maximise efficiency and comfort.

### Clinical applicability

**Fig. 6:**
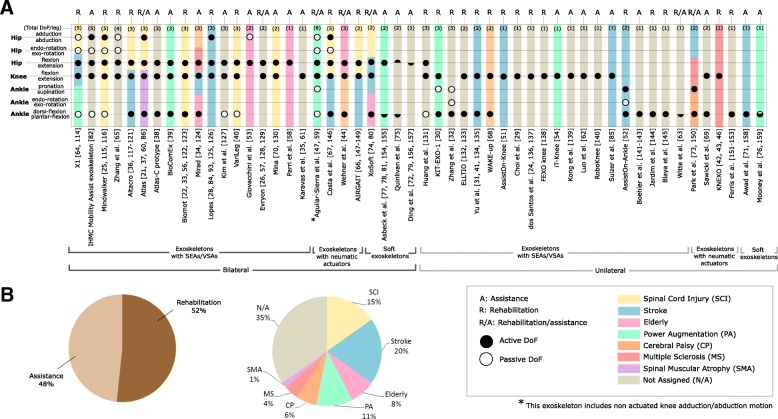
Classification of 52 lower limb exoskeletons^11^. **a** The robots are classified into function (rehabilitation/assistance), number of active and passive DoF, targeted pathologies, actuation system (SEA/VSA, pneumatic actuator) and configuration (unilateral/bilateral). **b** A breakdown of the individual system’s information is shown

Exoskeletons for rehabilitation or assistance are characterized by a large variety of number and typology of DoF (see Fig. 6-a). Active DoF^9^ are usually needed to substitute or compensate the joint torques necessary for body transport. Passive DoF^10^ may also be included to cope with other biomechanical functions, such as shock absorption or weight bearing [40, 82].

In patients affected by Spinal Cord Injury (SCI), the type of support depends on the level of the lesion and its severity [83]. When the lesion is complete, an exoskeleton must substitute the entire lower limb motor function and support the whole body weight. Lower limb exoskeletons for SCI patients are usually bilateral and have two or more DoF per leg (see e.g. [22, 25, 36, 40, 64, 66, 67] in Fig. 6). Exoskeletons for post-stroke individuals should compensate for the incorrect/insufficient lower limb motion. Therefore, actuation can be unilateral or bilateral. Most of bilateral exoskeletons for post-stroke individuals include more than 2 DoF per leg [34, 36, 64, 66, 67, 84], whereas unilateral devices include only one or two DoF [41, 51, 52, 71, 73, 74, 85]. While most of the reviewed exoskeletons focus on stroke, SCI, or older adults, other solutions address other neurological or non—neurological pathologies, such as cerebral palsy [44, 68, 73], multiple sclerosis [34, 42, 73], spinal muscular atrophy [86] or are used for strength augmentation [30, 39, 54, 59, 64, 76, 78].

Gait disorders and lower limb impairments are also related to ageing [87]. In this scenario, lower limb exoskeletons are used to compensate or augment motor function, and tend to be bilateral [34, 44, 53, 58]. The majority of exoskeletons were designed for adults, whereas only three devices were specifically designed for children [38, 60, 68].

## Discussion

### Exoskeletons with compliant actuation

Solutions based on SEAs and VSAs are highly heterogeneous, resulting in a strong non-linear relationship between maximum torque and weight. This, together with the scarce of technical data reported, make it difficult to identify one best design option. Most of the actuators are the result of a trial-and-error design process, based on past experience and the specific needs of the application. The selection of the elastic component type and spring stiffness remains a major open challenge for SEA or VSA designs. From the control point of view, higher stiffness is preferred in order to increase the bandwidth of the system [15]. However, this can hinder the intrinsic adaptability offered by such systems. The choice of the appropriate stiffness has implications on safety. In rigid actuators, including a compliant element could have the advantage of improving the safety, e.g. in unexpected impacts between the user and the device [27]. Nevertheless, this does not always hold true as the energy stored in a spring can be suddenly released during impacts or misuse of the device, generating unexpected and unsafe reactions [88]. The selection of the optimal spring stiffness should involve a theoretical analysis and experimental validation for the specific application [16, 25, 33]. The compliant/rigid dichotomy has to be considered at the start of the project and the actuation type should be carefully selected when designing a compliant exoskeleton. For instance, when testing actuation bandwidth, well-defined and standardised experiments (e.g. with fixed loads) should be performed in order to contrast with other actuators’ results.

VSAs have been proposed as a solution to make robots more adaptable to user’s residual abilities and biomechanical restrictions [19]. Nevertheless, this actuation concept requires the inclusion of extra mechanisms or motors for on-line stiffness modulation, thus resulting in considerable increases in weight and complex designs. The benefits of VSAs are still to be shown in view of these disadvantages.

We found several compliant exoskeletons with pneumatic actuators in the literature. Their dependence on off-board air supply restricts the ambulatory applications of these exoskeletons. Most of the publications did not include sufficient technical details with respect to, for instance, air flow rate, pressure level for contraction and expansion and other technical characteristics of the artificial muscle (i.e. diameter, length). This information, in our opinion, would have been beneficial to compare the different solutions and therefore help the convergence on successful design strategies.

Modularity, defined as the application of the same actuator to different active DoF, is a common practice in exoskeleton design as a strategy to reduce costs as well as effort in manufacturing, development and tuning [25]. While simplifying the mechanical design, this approach often results in oversized actuators. A promising approach to improve the power-to-weight ratio is to apply modularity only on the actuation principle, while optimizing the mechanical design of the actuator to the specific torque requirements for a given joint [22, 36].

The structure’s total weight and weight distribution have considerable impact on the functional performance and metabolic consumption [89]. Simulation-based optimization demonstrated to be a practical tool to reach lower weights while maintaining high mechanical properties [90]. Misalignments are more likely to happen with rigid structures, with negative effects on functionality, comfort and user’s safety [91]. Different solutions to solve these problems have been reviewed in a number of previous works [50, 51, 53, 54, 56]. The introduction of multiple passive DoF is still the most effective option. Nevertheless, this solution adds extra weight to the exoskeleton and tends to complicate the structure and its control due to increased inertia and friction [48, 92]. Some successful exoskeletons with rigid structure improve user-exoskeleton interaction by reducing metabolic cost and not considering extra passive DoF addition [69, 85]. Further research in this line is needed to find optimal solutions.

Inappropriate physical contact between the user and the robot is an issue potentially affecting pain and discomfort [93, 94], and inefficient or inappropriate, e.g. delayed, transmission of forces [95]. Attachment design has to consider the inherent non-linear viscoelastic properties of human soft tissues, such as tendons, ligaments and skin [96, 97]. Compliant actuation adds complexity to these interaction dynamics [33]. In this regard, models for predicting interaction forces are a promising approach [98]. Humidity and temperature changes occurring in the surfaces of the skin in contact with the interface lead to risk of skin damage [49], and should be carefully evaluated, in particular when a prolonged use of the device is envisioned. The anatomical fit of the robot is another challenge. The user-specific approach used in the upper limb exoskeleton developed by Chiri et al. [99] is a good example on how to personalize the interface.

### Soft exoskeletons

Soft exoskeletons present three main advantages with respect to compliant exoskeletons with rigid structures. First, the cable transmission allows optimizing the number and location of the actuators, with direct effects on the weight and inertia of the device. Second, the soft structure strongly reduce the misalignments and kinematic incompatibility between user and device [81]. Third, the soft structures are usually thin and suitable to be worn under user’s clothes [71], which is appropriate for usability. However, such design solutions entail inevitable drawbacks. Cable transmission requires actuators to be placed either off-board, preventing ambulatory use, or on-board, compelling the user to carry a backpack. Cables and textiles routed between the actuator and the targeted joint generate undesirable loads to the joints along the path [100]. The non-linear viscoelastic properties of soft element result in control bandwidth reduction and inefficient power transmission [78]. The absence of rigid structure through soft element is usually associated to shear forces and soft tissues deformation, which contribute to reduce user comfort [101]. Additionally, the inability to passively support the user weight limits the use of soft exoskeleton in patients with minimal residual motor abilities. Some promising approaches to solve these problems rely on modelling the interaction dynamics between soft structures and user to improve control [101], increasing the stiffness of the textiles to maximize power transfer [78], and designing compatible sensors able to accurately and reliably measure joint kinematics and kinetics of soft structures [100, 102]. From an ergonomics standpoint, the soft structures of these exoskeletons have to be preloaded against the user body to limit undesirable motions [78]. As a result, the structure has to be tightly dimensioned on user height and morphology.

### Fully compliant exoskeletons

Only two out of the 52 reviewed exoskeletons included a combination of soft structure and compliant actuation. This choice is promising, and likely to lead to lighter devices with higher torque actuation performance with respect to current solutions (Fig. 4-B) [44, 73]. Nevertheless, due to the very few works identified in the literature, more research is needed to define the actual benefits and drawbacks of this option.

### Clinical applicability

Choosing the right actuation type, the structure, and the physical interfaces and number of DoFs is a hard problem, which strongly depends on the application. Compliant exoskeletons with SEAs or VSAs are the most popular choice for ambulatory solutions for activities of daily living. This justifies the huge number of exoskeletons with this actuation type and the broad range of different existing designs. Pneumatic and soft exoskeletons with off-board actuators are preferred for non-ambulatory gait rehabilitation and assistance applications in the clinical setting. Soft structures are usually preferred in exoskeletons for gait restoration for individuals who still retain some walking ability (Fig. 6) [71]. Conversely, in people with strong body weight support needs, exoskeletons with rigid structures are the preferred option [25, 40].

A challenging issue is the ergonomic adaptation of exoskeletons to a wide range of patients within a clinical environment. In this case, designers have to use up-to-date anthropometric databases when defining the dimensions of the structures and interfaces. Scanning patient’s limbs and obtaining the brace with additive manufacturing techniques is a promising, and low cost, solution [103]. A critical issue with 3D printing technology is the durability of the materials [104]. Thus, the study of materials and manufacturing techniques represent, in our opinion, a promising direction in order to get long-life and comfortable interfaces. However, how to address ergonomics and comfort is an unclear issue that has to be seriously taken into account in the design of exoskeletons. Under the DoF perspective, we found a strong variability across the reviewed works. This choice depends on several factors, mostly driven by the specific user needs, such as the level of reduced mobility of the user or the functional purpose of the exoskeleton, e.g. rehabilitation or assistance. Nevertheless, we could not find a clear relationship between these factors. Further research is needed to understand this issue.

A particularly relevant problem related to the use of exoskeletons in rehabilitation or assistance is their effect on balance. Users’ balance may be compromised when using ambulatory unilateral or bilateral exoskeletons, due to the weight of the device and its behaviour. In addition, the loss of walking functions in most patients is frequently associated with balance disorders [105]. As a result, ambulatory exoskeletons are normally used in combination with crutches [25, 40, 70, 82]. The use of crutches may improve user’s self-confidence, serve as a feedback tool, and reduce the risks of falls [82]. In clinical/research settings, non-ambulatory exoskeleton are usually supported with treadmill-based structures, standing structures or safety harness [26, 36, 38, 42, 59, 60, 65–67, 71, 84, 85]. Apart from the inclusion of these safety devices, balance is a topic that has been largely overlooked in the exoskeleton literature, and should be seriously considered in the future, both from the assessment and control point of view.

The studies carried out to date have seldom included the user’s subjective perception of the exoskeleton in their evaluations. Including the users’ opinion can shed light to the design process, in particular the design of the structure and the interface [74]. Satisfaction scales, such as Borg and QUEST scales, can be used to this end [106]. These scales have proven to have high reliability and validity [107]. Schiele studied subjective performance metrics and used a NASA TLX questionnaire to evaluate comfort in subjects using an upper limb exoskeleton [108].

## Conclusions

In this review, we described and compared the mechanical design choices of 52 lower limb compliant exoskeletons. We have limited our analysis to solutions that included mechanical compliant properties in the actuation and/or structure, excluding all those devices whose compliant behaviour is obtained by control strategies, namely virtual or active compliance. We focused on three main technical aspects, i.e. the actuators, the structure, and the interface attachment components. Compliant actuators are still heterogeneous and there are not clear established criteria for their design. The robotics community would highly benefit from specific guidelines that can speed up the development and the uptake of these technologies. Promising directions go in favour of more compact and personalized actuators, dimensioned on joint- and patient-specific torque requirements. These solutions will require extra efforts in terms of mechanical design with respect to traditional modular approaches, but are likely to produce more lightweight and efficient solutions. The choice of the structure material and morphology is also a crucial factor, with important effects on comfort and actuation principles.

The physical interface between human and exoskeleton is a factor that has been particularly overlooked by current research. It should be seriously considered in the future to account for comfort, adaptation and efficiency problems. The soft exoskeleton technology, despite having several potential advantages over classic rigid approaches, needs to be further analysed in order to become a useful tool for rehabilitation and assistive applications.

Under the DoF perspective, we found a great heterogeneous picture, with unclear correlation between technical solutions and pathologies addressed. Future efforts should be devoted in clarifying whether and how the number, type (active/passive) and distribution of DoF affects the performance of the different targeted populations.

Finally, we observed a clear lack of technical information, metrics and benchmarks of performance across the reviewed literature. This input would be of great help to evaluate and compare the different devices on a standardized basis [109, 110]. In this respect, the community should support and encourage homogeneity in technical reporting, to allow replicability and truly comparison. As a first step in this direction, we generated available data sheets (see Additional file 1), in which we gathered the main available technical information on each of the reviewed exoskeletons.

## Additional file

Additional file 1: Compliant lower limb exoskeletons: A comprehensive review on mechanical design principles. Technical characteristics of 52 compliant exoskeletons reviewed in the article including information about their actuation system, structure, interface attachment components, applicability, control strategies and related publications (PDF 18064 kb)

## Abbreviations

CP: Cerebral Palsy
DoF: Degrees of Freedom
EDM: Electrical Discharge Machining
FEA: Finite Elements Analysis
MS: Multiple Sclerosis
PA: Power Augmentation
PAM: Powered Artificial Muscle
PPAM: Pleated Pneumatic Artificial Muscle
SCI: Spinal Cord Injury
SEA: Series Elastic Actuator
SMA: Spinal Muscular Atrophy
VSA: Variable Stiffness Actuator

## Footnotes

1 Exoskeleton: Mechanical system worn by humans to augment, complement or substitute the function of natural limbs which work in parallel with the human body [49]. The term ‘Wearable robot’ is commonly used interchangeably.

2 Structure: Mechanical components that transmit forces from the actuators to the interface attachment components of the exoskeleton. Structure can be composed of rigid and/or non-rigid materials.

3 Interface attachment components: Mechanical components that transmit forces between the structure of the exoskeleton and the user [101]. They are usually composed of rigid or semi-rigid braces or cuffs ensured to body segment through belts and/or straps.

4 Soft exoskeleton: Exoskeleton composed of non-rigid structure (i.e. textiles, straps, sleeves) to interface with the human body [111]. The term ‘soft exosuit’ is commonly used interchangeably.

5 Compliant actuation: Movement of the robotic system in order to perform its function; accomplished by some components with compliant properties (i.e. springs, custom made compliant mechanisms, etc.). The compliant actuator allows deviations from its equilibrium position [112].

6 Backdrivable actuator: Actuation system with low impedance behaviour when not powered [15, 24].

11 References [114–159] include publications related to the 52 lower limb exoskeletons reviewed in this article that are not mentioned within the main body of the manuscript but appear in the figures.

7 Dress on: Put in contact. Attach the robot to the user prior to the exoskeleton is turned on.

8 Dress off: Interrupt the contact. Disengage the robot of the user after the exoskeleton is turned off.

9 Active DoF: DoF that requires a power supply to be moved. The term ‘powered DoF’ is commonly used interchangeably.

10 Passive DoF: DoF that does not require power supply to be moved. Commonly used mechanisms include springs, elastic elements and dampers [113].
