# Supplementary material for: Compliant lower limb exoskeletons: a comprehensive review on mechanical design principles
Source: J Neuroeng Rehabil. 2019 May 9;16:55. doi: 10.1186/s12984-019-0517-9 (PMC6506961; doi:10.1186/s12984-019-0517-9)
Supplement: Supplementary file 1 — Compliant lower limb exoskeletons: A comprehensive review on mechanical design principles. Technical characteristics of 52 compliant exoskeletons reviewed in the article including information about their actuation system, structure, interface attachment components, applicability, control strategies and related publications (PDF 18064 kb) [file 12984_2019_517_MOESM1_ESM.pdf]

Data sheets

Compliant lower limb exoskeletons: A comprehensive review on mechanical design principles

List of acronyms

**Ankle_DP**	Ankle Dorsi/Plantar-flexion
**Ankle_EE**	Ankle Endo/Exo-rotation
**Ankle_P**	Ankle Plantar-flexion
**Ankle_PS**	Ankle Pronation/Supination
**ARES**	Adjustable Rigidity and Embedded Sensor
**CP**	Cerebral Palsy
**C-DSSAS**	Compact Dual Spiral Spring Actuation System
**CompAct-ARS**	CompAct Actuator with Reconfigurable Stiffness
**cRSEA**	compact Rotary Series Elastic Actuator
**Hip_AA**	Hip Ab/Adduction
**Hip_E**	Hip Extension
**Hip_EE**	Hip Endo/Exo-rotation
**Hip_F**	Hip Flexion
**Hip_FE**	Hip Flexion/Extension
**Knee_FE**	Knee Flexion/Extension
**MACCEPA**	Mechanically Adjustable Compliance and Controllable Equilibrium Position Actuator
**MS**	Multiple Sclerosis
**N/A**	Not Available
**PA**	Power Augmentation
**PAM**	Pneumatic Artificial Muscle
**PPAM**	Pleated Pneumatic Artificial Muscle
**RSEA**	Rotary Series Elastic Actuator
**SCI**	Spinal Cord Injury
**SMA**	Spinal Muscular Atrophy
**SEA**	Series Elastic Actuator
**VSA**	Variable Stiffness Actuator

Actuators

Type	SEA
Location	On-board
Power	N/A
Maximum torque	N/A
Maximum speed	N/A
Bandwidth	N/A
Weight	N/A
Modular actuators	N/A
Actuator specific characteristics	
Spring typology	N/A
Spring stiffness	N/A
Actuator stiffness	N/A

Structure & interface attachment components

Allowable wearer maximum weight	N/A
Allowable wearer height range	N/A
Anthropomorphic considerations	A range of user spanning from the 5th to 95th percentile was considered.
Configurable dimensions	· Pelvis width<br>· Hip axis-knee axis distance<br>· Knee axis-ankle axis distance
Structure typology	Rigid structure
Braces/segment	1

Applicability & general characteristics

Target user	Adults
Application	Rehabilitation (SCI/stroke)/ PA
Unilateral/bilateral	Bilateral
Set up	Ambulatory
Exoskeleton weight	26 kg
Total DoFs	5 (Hip_AA, Hip_EE, Hip_FE, Knee_FE, Ankle_DP)
Actuated DoFs	2 (Hip_FE, Knee_FE)
Walking speed	N/A

Control

Control strategies	· Impedance control<br>· Position control<br>· Torque control<br>· Zero assistance control

Electric power supply

Battery autonomy	N/A

Related publications

[1] Rea R, Beck C, Rovekamp R. X1 : A Robotic Exoskeleton for In - Space Countermeasures. In: AIAA SPACE 2013 Conference and Exposition. San Diego (USA); 2013. p. 5510. doi:10.2514/6.2013-5510.

[2] He Y, Nathan K, Venkatakrishnan A, Rovekamp R, Beck C, Francisco GE, et al. An Integrated Neuro-Robotic Interface for Stroke Rehabilitation using the NASA X1 Powered Lower Limb Exoskeleton. In: 36th Annual International Conference of the IEEE EMBS. Chicago (USA); 2014. p. 3985–8. doi:10.1109/EMBC.2014.6944497.

IHMC Mobility Assist exoskeleton

Actuators

Type	RSEA
Location	On-board
Power	N/A
Maximum torque	80 Nm
Maximum speed	6.80 rad/s
Bandwidth	15 Nm-10 Hz; <15 Nm-30 Hz
Weight	N/A
Modular actuators	✓
Actuator specific characteristics	
Spring typology	Linear spring, Bowden cable
Spring stiffness	N/A
Actuator stiffness	N/A

Structure & interface attachment components

Allowable wearer maximum weight	N/A
Allowable wearer height range	N/A
Anthropomorphic considerations	N/A
Configurable dimensions	N/A
Structure typology	Rigid structure
Braces/segment	Combined solution (2 thigh, 1 shank)

Applicability & general characteristics

Target user	Adults
Application	Assistance
Unilateral/bilateral	Bilateral
Set up	Ambulatory (crutches/walker)
Exoskeleton weight	N/A
Total DoFs	5 (Hip_AA, Hip_EE, Hip_FE, Knee_FE, Ankle_DP)
Actuated DoFs	3 (Hip_AA, Hip_FE, Knee_FE)
Walking speed	N/A

Control

Control strategies	· Torque/force control<br>· Position control<br>· Zero impedance control

Electric power supply

Battery autonomy	N/A

Related publications

[1] Missel M, Pra JE, Ne PD. Development of the IHMC Mobility Assist Exoskeleton. In: 2009 IEEE International Conference on Robotics and Automation (ICRA). Kobe (Japan); 2009. doi:10.1109/ROBOT.2009.5152394.

Actuators

Type	SEA
Location	On-board
Power	1 kW (actuator)
Maximum torque	100 Nm
Maximum speed	N/A
Bandwidth	100 Nm-6.7 Hz; 2 Nm-38.2 Hz
Weight	2.90 kg
Modular actuators	✓
Actuator specific characteristics	
Spring typology	Custom made disc-shaped torsional spring
Spring stiffness	820 Nm/rad
Actuator stiffness	N/A

Structure & interface attachment components

Allowable wearer maximum weight	100 kg
Allowable wearer height range	153-188 cm
Anthropomorphic considerations	Anatomical measures of 90% of the European adult population were considered (user height and hip width)
Configurable dimensions	· Pelvis width<br>· Hip axis-knee axis distance<br>· Knee axis-ankle axis distance
Structure typology	Rigid structure
Braces/segment	1

Applicability & general characteristics

Target user	Adults
Application	Rehabilitation (SCI)
Unilateral/bilateral	Bilateral
Set up	Ambulatory (crutches)
Exoskeleton weight	28 kg
Total DoFs	5 (Hip_AA, Hip_EE, Hip_FE, Knee_FE, Ankle_DP)
Actuated DoFs	3 (Hip_AA, Hip_FE, Knee_FE)
Walking speed	0.80 m/s

Control

Control strategies	· Variable impedance control<br>· Torque control

Electric power supply

Battery autonomy	N/A

Related publications

[1] Wang S, Wang L, Meijneke C, Asseldonk E Van, Hoellinger T, Cheron G, et al. Design and Control of the MINDWALKER Exoskeleton. IEEE Trans Neural Syst Rehabil Eng. 2015;23:277–86. doi:10.1109/TNSRE.2014.2365697.
[2] Wang S, Meijneke C, van der Kooij H. Modeling, design, and optimization of Mindwalker series elastic joint. In: 2013 IEEE International Conference on Rehabilitation Robotics (ICORR). Seattle (USA); 2013. doi:10.1109/ICORR.2013.6650381.
[3] Wang L, Wang S, Asseldonk EHF Van, Kooij H Van Der. Actively Controlled Lateral Gait Assistance in a Lower Limb Exoskeleton. In: 2013 IEEE/RSJ International Conference on Intelligent Robots and Systems (IROS). Tokyo (Japan); 2013.

doi:10.1109/IROS.2013.6696467.

Zhang et al. [1]

Actuators

Type	SEA
Location	On-board
Power	N/A
Maximum torque	22 Nm
Maximum speed	N/A
Bandwidth	N/A
Weight	N/A
Modular actuators	✘
Actuator specific characteristics	
Spring typology	N/A
Spring stiffness	N/A
Actuator stiffness	N/A

Structure & interface attachment components

Allowable wearer maximum weight	75 kg
Allowable wearer height range	N/A
Anthropomorphic considerations	N/A
Configurable dimensions	N/A
Structure typology	Rigid structure
Braces/segment	1

Applicability & general characteristics

Target user	Adults
Application	Rehabilitation
Unilateral/bilateral	Bilateral
Set up	Safety harness
Exoskeleton weight	30
Total DoFs	4 (Hip_AA, Hip_EE, Hip_FE, Knee_FE)
Actuated DoFs	2 (Hip_FE, Knee_FE)
Walking speed	1.3 m/s

Control

Control strategies	Position control

Electric power supply

Battery autonomy	N/A

Related publications

[1] Zhang C, Liu G, Li C, Zhao J, Yu H, Zhu Y. Development of a lower limb rehabilitation exoskeleton based on real-time gait detection and gait tracking. Adv Mech Eng. 2016;8:1–9. doi:10.1177/1687814015627982.

Actuators

Type	MACCEPA (VSA)
Location	On-board
Power	180 W (actuator)
Maximum torque	40 Nm
Maximum speed	5.80 rad/s
Bandwidth	3 Hz
Weight	2.40 kg
Modular actuators	✓
Actuator specific characteristics	
Spring typology	Linear spring
Spring stiffness	28 N/mm
Actuator stiffness	110 Nm/rad

Structure & interface attachment components

Allowable wearer maximum weight	75 kg
Allowable wearer height range	N/A
Anthropomorphic considerations	N/A
Configurable dimensions	N/A
Structure typology	Rigid structure
Braces/segment	Combined solution (2 thigh, 1 shank)

Applicability & general characteristics

Target user	Adults
Application	Rehabilitation (SCI/stroke)
Unilateral/bilateral	Bilateral
Set up	Treadmill-based (support structure)
Exoskeleton weight	N/A
Total DoFs	3 (Hip_FE, Knee_FE, Ankle_DP)
Actuated DoFs	3 (Hip_FE, Knee_FE, Ankle_DP)
Walking speed	N/A

Control

Control strategies	· Force control<br>· Position control<br>· Torque control<br>· Zero torque control<br>· Impedance control

Electric power supply

Battery autonomy	N/A

Related publications

[1] Brackx B, Grosu V, Ham RVAN, Damme MVAN, Vanderborght B, Lefeber D. Design of the gait rehabilitation robot ALTACRO: a powered exoskeleton using compliant actuation. In: 5th International Workshop on Human-Friendly Robotics. Brussels (Belgium); 2012.
[2] Damme VAN, Vanderborght B. Electronic hardware architecture of step rehabilitation robot ALTACRO. In: 9th National Congress on Theoretical and Applied Mechanics. Brussels (Belgium); 2012. p. 9–10.
[3] Grosu V, Rodriguez Gerrero C, Brackx B, Grosu S, Vanderborght B, Lefeber D. Instrumenting Complex Exoskeletons for Improved Human-Robot Interaction. IEEE Instrum Meas Mag. 2015;18:5–10. doi:10.1109/MIM.2015.7271219.

[4] Junius K, Cherelle P, Brackx B, Geeroms J, Schepers T, Vanderborght B, et al. On the use of adaptable compliant actuators in prosthetics, rehabilitation and assistive robotics. In: 9th International Workshop on Robot Motion and Control. Wasowo (Poland); 2013.
[5] Cherelle P, Grosu V, Beyl P, Mathys A, Ham R Van, Damme M Van, et al. The MACCEPA Actuation System as Torque Actuator in the Gait Rehabilitation Robot ALTACRO. In: 3rd IEEE RAS & EMBS International Conference on Biomedical Robotics and Biomechatronics (BioRob). Tokyo (Japan); 2010. p. 27–32. doi:10.1109/BIOROB.2010.5627030.
[6] Grosu V, Rodriguez-Guerrero C, Grosu S, Vanderborght B, Lefeber D. Design of Smart Modular Variable Stiffness Actuators for Robotic-Assistive Devices. IEEE/ASME Trans Mechatronics. 2017;22:1777–85. doi:10.1109/TMECH.2017.2704665.

Actuators

Type	ARES (VSA)
Location	On-board
Power	90 W (motor)
Maximum torque	76 Nm
Maximum speed	2.50 rad/s
Bandwidth	N/A
Weight	0.90 kg
Modular actuators	✗
Actuator specific characteristics	
Spring typology	Linear spring
Spring stiffness	N/A
Actuator stiffness	260.50 Nm/rad

Structure & interface attachment components

Allowable wearer maximum weight	35 kg
Allowable wearer height range	100-135 cm
Anthropomorphic considerations	N/A
Configurable dimensions	· Hip axis-knee axis distance<br>· Knee axis-ankle axis distance
Structure typology	Rigid structure
Braces/segment	1

Applicability & general characteristics

Target user	Children
Application	Assistance/rehabilitation (SCI, SMA)
Unilateral/bilateral	Bilateral
Set up	Special standing frame (walker frame)
Exoskeleton weight	14 kg
Total DoFs	4 (Hip_AA, Hip_FE, Knee_FE, Ankle_DP)
Actuated DoFs	4 (Hip_AA, Hip_FE, Knee_FE, Ankle_DP)
Walking speed	0.50 m/s

Control

Control strategies	N/A

Electric power supply

Battery autonomy	2.5 hours

Related publications

[1] Sanz-Merodio D, Cestari M, Arevalo JC, Garcia E. A lower-limb exoskeleton for gait assistance in quadriplegia. In: 2012 IEEE International Conference on Robotics and Biomimetics. Guangzhou (China); 2012. p. 122–7. doi:10.1109/ROBIO.2012.6490954.
[2] Cestari M, Sanz-Merodio D, Arevalo JC, Garcia E. An Adjustable Compliant Joint for Lower-Limb Exoskeletons. IEEE/ASME Trans Mechatronics. 2015;20:889–98. doi:10.1109/TMECH.2014.2324036.
[3] Cestari M, Sanz-Merodio D, Arevalo JC, Garcia E. ARES, a variable stiffness actuator with embedded force sensor for the ATLAS exoskeleton. Ind Rob. 2014;41:518–26. doi:10.1108/IR-06-2014-0350.
[4] Ganguly A, Puyuelo G, Goñi A, Garces E, Garcia E. Wearable pediatric gait exoskeleton- a feasibility study*. In: 2018 IEEE/RSJ International Conference on Intelligent Robots and Systems (IROS). Madrid (Spain); 2018. p. 4667–72.

ATLAS - C prototype

Actuators

Type	ARES (SEA)*, ARES-XL (VSA)**<br>(*=hip actuator, **=knee/ankle actuator)
Location	On-board
Power	90 W* (motor), N/A**
Maximum torque	76 Nm*, N/A**
Maximum speed	2.50 rad/s*, N/A**
Bandwidth	N/A
Weight	0.90 kg*, N/A**
Modular actuators	✘
Actuator specific characteristics	
Spring typology	Linear spring
Spring stiffness	16.80 N/mm**
Actuator stiffness	260.50 Nm/rad*

Structure & interface attachment components

Allowable wearer maximum weight	N/A
Allowable wearer height range	N/A
Anthropomorphic considerations	N/A
Configurable dimensions	N/A
Structure typology	Rigid structure
Braces/segment	N/A

Applicability & general characteristics

Target user	Children
Application	Assistance
Unilateral/bilateral	Bilateral
Set up	Special standing frame (walker frame)
Exoskeleton weight	N/A
Total DoFs	3 (Hip_FE, Knee_FE, Ankle_DP)
Actuated DoFs	3 (Hip_FE, Knee_FE, Ankle_DP)
Walking speed	N/A

Control

Control strategies	N/A

Electric power supply

Battery autonomy	N/A

Related publications

[1] Cestari M, Sanz-Merodio D, Garcia E. Preliminary Assessment of a Compliant Gait Exoskeleton. Soft Robot. 2017. doi:10.1089/soro.2016.0070.

BioComEx

Actuators

Type	SEA*, VSA** (*=hip and knee actuators, **=ankle actuator)
Location	On-board
Power	N/A
Maximum torque	N/A
Maximum speed	N/A
Bandwidth	N/A
Weight	N/A
Modular actuators	✘
Actuator specific characteristics	
Spring typology	Linear spring**
Spring stiffness	N/A
Actuator stiffness	N/A

Structure & interface attachment components

Allowable wearer maximum weight	N/A
Allowable wearer height range	N/A
Anthropomorphic considerations	N/A
Configurable dimensions	· Hip axis-knee axis distance<br>· Knee axis-ankle axis distance<br>· Foot length
Structure typology	Rigid structure
Braces/segment	1

Applicability & general characteristics

Target user	Adults
Application	Assistance (PA)
Unilateral/bilateral	Bilateral
Set up	Ambulatory
Exoskeleton weight	N/A
Total DoFs	3 (Hip_FE, Knee_FE, Ankle_DP)
Actuated DoFs	3 (Hip_FE, Knee_FE, Ankle_DP)
Walking speed	N/A

Control

Control strategies	N/A

Electric power supply

Battery autonomy	N/A

Related publications

[1] Baser O, Kizilhan H, Kilic E. Mechanical Design of a Biomimetic Compliant Lower Limb Exoskeleton (BioComEx). In: 2016 International Conference on Autonomous Robot Systems and Competitions (ICARSC). Braganca (Portugal); 2016. p. 60–5. doi:10.1109/ICARSC.2016.51.

Actuators

Type	MACCEPA (VSA)<br>(*=knee actuator, **=ankle actuator)
Location	On-board
Power	N/A
Maximum torque	50 Nm
Maximum speed	N/A
Bandwidth	N/A
Weight	1.18 kg**
Modular actuators	✗
Actuator specific characteristics	
Spring typology	Linear spring
Spring stiffness	70 N/mm*
Actuator stiffness	N/A

Struture & interface attachment components

Allowable wearer maximum weight	80 kg
Allowable wearer height range	N/A
Anthropomorphic considerations	Total leg segment adjustability in between 0.35 and 0.45 m.
Configurable dimensions	· Pelvis width<br>· Hip axis-knee axis distance<br>· Knee axis-ankle axis distance
Structure typology	Rigid structure
Braces/segment	2

Applicability & general characteristics

Target user	Adults
Application	Rehabilitation (SCI)
Unilateral/bilateral	Bilateral
Set up	Ambulatory
Exoskeleton weight	19.40 kg
Total DoFs	3 (Hip_FE, Knee_FE, Ankle_DP)
Actuated DoFs	3 (Hip_FE, Knee_FE, Ankle_DP)
Walking speed	N/A

Control

Control strategies	Torque control

Electric power supply

Battery autonomy	N/A

Related publications

[1] Bacek T, Moltedo M, Langlois K, Prieto GA, Sanchez-Villamanan MC, Gonzalez-Vargas J, et al. BioMot exoskeleton — Towards a smart wearable robot for symbiotic human-robot interaction. In: 2017 International Conference on Rehabilitation Robotics (ICORR). London (UK); 2017. p. 1666–71. doi:10.1109/ICORR.2017.8009487.
[2] Bacek T, Unal R, Moltedo M, Junius K, Cuypers H, Vanderborght B, et al. Conceptual Design of a Novel Variable Stiffness Actuator for Use in Lower Limb Exoskeletons. In: 2015 IEEE International Conference on Rehabilitation Robotics (ICORR). Singapore (Singapore); 2015. doi:10.1109/ICORR.2015.7281263.
[3] Moltedo M, Bacek T, Junius K, Vanderborght B, Lefeber D. Mechanical Design of a Lightweight Compliant and Adaptable

Active Ankle Foot Orthosis. In: 6th IEEE RAS/EMBS International Conference on Biomedical Robotics and Biomechatronics (BioRob). Singapure (Singapure); 2016. p. 1224–9. doi:10.1109/BIOROB.2016.7523798.
[4] Bacek T, Moltedo M, Gonzalez-Vargas J, Asin Prieto G, Sanchez-Villamañan MC, Moreno JC, et al. The new generation of compliant actuators for use in controlable bio-inspired wearable robots. In: Wearable robotics: challenges and trends; proceedings of the 2nd International Symposium on Wearable Robotics (WeRob2016). La Granja (Segovia), Spain; 2016. p. 255–60.
[5] Moltedo M, Bacek T, Langlois K, Junius K, Vanderborght B, Lefeber D. Design and experimental evaluation of a lightweight, high-torque and compliant actuator for an active ankle foot orthosis. In: 2017 International Conference on Rehabilitation Robotics (ICORR). London (UK); 2017. p. 283–8. doi:10.1109/ICORR.2017.8009260.

Actuators

Type	MACCEPA (VSA)
Location	On-board
Power	N/A
Maximum torque	15 Nm
Maximum speed	N/A
Bandwidth	N/A
Weight	1.40 kg
Modular actuators	✓
Actuator specific characteristics	
Spring typology	Linear spring
Spring stiffness	15 N/mm
Actuator stiffness	28.07-81.36 Nm/rad

Structure & interface attachment components

Allowable wearer maximum weight	80 kg
Allowable wearer height range	165-190 cm
Anthropomorphic considerations	N/A
Configurable dimensions	N/A
Structure typology	Rigid structure
Braces/segment	1

Applicability & general characteristics

Target user	Adults
Application	Assistance (Elderly/MS/stroke/CP)
Unilateral/bilateral	Bilateral
Set up	Ambulatory
Exoskeleton weight	13 kg
Total DoFs	3 (Hip_FE, Knee_FE, Ankle_DP)
Actuated DoFs	3 (Hip_FE, Knee_FE, Ankle_DP)
Walking speed	N/A

Control

Control strategies	· Assistance-as-needed control<br>· Position control

Electric power supply

Battery autonomy	N/A

Related publications

[1] Junius K, Brackx B, Grosu V, Cuypers H, Geeroms J, Moltedo M, et al. Mechatronic Design of a Sit-to-Stance Exoskeleton. In: 5th IEEE RAS & EMBS International Conference on Biomedical Robotics and Biomechatronics (BioRob). Sao Paulo (Brazil); 2014. p. 945–50. doi:10.1109/BIOROB.2014.6913902.

[2] Brackx B, Geeroms J, Vantilt J, Grosu V, Junius K, Cuypers H, et al. Design of a Modular Add-on Compliant Actuator to Convert an Orthosis into an Assistive Exoskeleton. In: 5th IEEE RAS & EMBS International Conference on Biomedical Robotics and Biomechatronics (BioRob). Sao Paulo (Brazil); 2014. doi:10.1109/BIOROB.2014.6913824.

Actuators

Type	SEA<br>(*=Hip/knee flexion-extension actuator,<br>**=Hip abduction-adduction actuator)
Location	On-board
Power	N/A
Maximum torque	100 Nm
Maximum speed	N/A
Bandwidth	11 Hz; 20 Hz
Weight	N/A
Modular actuators	✓
Actuator specific characteristics	
Spring typology	Bowden cable, linear spring
Spring stiffness	35.10 N/mm*, 57.20 N/mm**
Actuator stiffness	N/A

Structure & interface attachment components

Allowable wearer maximum weight	90 kg
Allowable wearer height range	N/A
Anthropomorphic considerations	N/A
Configurable dimensions	· Pelvis width<br>· Hip axis-knee axis distance
Structure typology	Rigid structure
Braces/segment	1

Applicability & general characteristics

Target user	Adult
Application	Rehabilitation (Stroke)
Unilateral/bilateral	Bilateral
Set up	Treadmill-based
Exoskeleton weight	N/A
Total DoFs	3 (Hip_AA, Hip_FE, Knee_FE)
Actuated DoFs	3 (Hip_AA, Hip_FE, Knee_FE)
Walking speed	0.75 m/s

Control

Control strategies	· Impedance control<br>· Position control<br>· Zero force/impedance control

Electric power supply

Battery autonomy	N/A

Related publications

[1] Veneman JF, Ekkelenkamp R, Kruidhof R, van der Helm FCT, van der Kooij H. Design of a Series Elastic and Bowdencable-Based Actuation System for Use As Torque-Actuator in Exoskeleton-Type Training Robots. In: 9th IEEE International Conference on Rehabilitation Robotics (ICORR). 2005. p. 496–9. doi:10.1109/ICORR.2005.1501150.
[2] Ekkelenkamp R, Veneman J, Kooij H Van Der. LOPES : a lower extremity powered exoskeleton. In: 2007 IEEE International Conference on Robotics and Automation (ICRA). Roma (Italy); 2007. p. 3132–3.
[3] Veneman JF, Kruidhof R, Hekman EEG, Ekkelenkamp R, Asseldonk EHF Van. Design and Evaluation of the LOPES Exoskeleton

Robot for Interactive Gait Rehabilitation. IEEE Trans Neural Syst Rehabil Eng. 2007;15:379–86. doi:10.1109/TNSRE.2007.903919.
[4] Kooij H Van Der, Veneman J, Ekkelenkamp R. Design of a compliantly actuated exo-skeleton for an impedance controlled gait trainer robot. In: 28th IEEE EMBS Annual International Conference. New York City (USA); 2006. p. 189–93. doi:10.1109/IEMBS.2006.259397.
[5] Veneman JF. Design and Evaluation of the Gait Rehabilitation Robot Lopes. University of Twente; 2007.

Kim et al. [1]

Actuators

Type	SEA
Location	On-board
Power	N/A
Maximum torque	N/A
Maximum speed	N/A
Bandwidth	N/A
Weight	N/A
Modular actuators	✓
Actuator specific characteristics	
Spring typology	Torsional spring
Spring stiffness	N/A
Actuator stiffness	N/A

Structure & interface attachment components

Allowable wearer maximum weight	N/A
Allowable wearer height range	N/A
Anthropomorphic considerations	Adult body measures were considered
Configurable dimensions	N/A
Structure typology	Rigid structure
Braces/segment	Combined solution (2 thigh, 1 shank)

Applicability & general characteristics

Target user	Adult
Application	Assistance
Unilateral/bilateral	Bilateral
Set up	Ambulatory
Exoskeleton weight	N/A
Total DoFs	3 (Hip_FE, Knee_FE, Ankle_DP)
Actuated DoFs	2 (Hip_FE, Knee_FE)
Walking speed	N/A

Control

Control strategies	Force control

Electric power supply

Battery autonomy	N/A

Related publications

[1] Kim S, Bae J. Development of a Lower Extremity Exoskeleton System for Human-Robot Interaction. In: 11th International Conference on Ubiquitous Robots and Ambient Intelligence (URAI). Kuala Lumpur (Malaysia); 2014. p. 132–5.

Actuators

Type	VSA*<br>(*=knee joint)
Location	On-board
Power	N/A
Maximum torque	89.60 Nm*
Maximum speed	2.04 rad/s*
Bandwidth	N/A
Weight	N/A
Modular actuators	✗
Actuator specific characteristics	
Spring typology	Linear spring*
Spring stiffness	109 N/mm
Actuator stiffness	380 Nm/rad

Structure & interface attachment components

Allowable wearer maximum weight	85 kg
Allowable wearer height range	175 – 190 cm
Anthropomorphic considerations	N/A
Configurable dimensions	Pelvis width: 34.5 - 40.0 cm.<br>Hip axis-knee axis distance: 43.5 - 49.5 cm<br>Knee axis-ankle axis distance: 38.0 - 44.0 cm
Structure typology	Rigid structure
Braces/segment	1

Applicability & general characteristics

Target user	Adults
Application	Assistance, Rehabilitation
Unilateral/bilateral	Bilateral
Set up	Ambulatory (crutches)
Exoskeleton weight	35 kg
Total DoFs	3 (Hip_FE, Knee_FE, Ankle_DP)
Actuated DoFs	2 (Hip_FE, Knee_FE)
Walking speed	N/A

Control

Control strategies	· Position control<br>· Stiffness control

Electric power supply

Battery autonomy	2 hours

Related publications

[1] Schrade SO, Dätwyler K, Stücheli M, Studer K, Türk D-A, Meboldt M, et al. Development of VariLeg, an exoskeleton with variable stiffness actuation: first results and user evaluation from the CYBATHLON 2016. J Neuroeng Rehabil. 2018;15:18. doi:10.1186/s12984-018-0360-4.

Giovaccini et al. [1]

Actuators

Type	SEA
Location	On-board
Power	N/A
Maximum torque	35 Nm
Maximum speed	N/A
Bandwidth	15 Hz
Weight	1.20 kg
Modular actuators	-
Actuator specific characteristics	
Spring typology	Custom made disc-shaped torsional spring
Spring stiffness	N/A
Actuator stiffness	100 Nm/rad

Structure & interface attachment components

Allowable wearer maximum weight	80 kg
Allowable wearer height range	N/A
Anthropomorphic considerations	N/A
Configurable dimensions	· Pelvis width<br>· Vertical position of the hip cuff
Structure typology	Rigid structure
Braces/segment	1

Applicability & general characteristics

Target user	Adults
Application	Assistance (Elderly)
Unilateral/bilateral	Bilateral
Set up	Ambulatory
Exoskeleton weight	4.20 kg
Total DoFs	2 (Hip_AA, Hip_FE)
Actuated DoFs	1 (Hip_FE)
Walking speed	N/A

Control

Control strategies	· Assistive control<br>· Torque control

Electric power supply

Battery autonomy	N/A

Related publications

[1] Giovacchini F, Vannetti F, Fantozzi M, Cortese M, Parri A, Yan T, et al. A light-weight active orthosis for hip movement assistance. Rob Auton Syst. 2014;73:123–34. doi:10.1016/j.robot.2014.08.015.

Actuators

Type	SEA
Location	On-board
Power	230 W (actuator)
Maximum torque	60 Nm
Maximum speed	N/A
Bandwidth	N/A
Weight	2.45 kg
Modular actuators	✓
Actuator specific characteristics	
Spring typology	Custom made disc-shaped torsional spring
Spring stiffness	250 Nm/rad
Actuator stiffness	N/A

Structure & interface attachment components

Allowable wearer maximum weight	80 kg
Allowable wearer height range	N/A
Anthropomorphic considerations	A range of the user spanning from the 5th to 95th percentile was considered
Configurable dimensions	Segment between the thigh brace and the shank brace
Structure typology	Rigid structure
Braces/segment	1

Applicability & general characteristics

Target user	Adults
Application	Rehabilitation/assistance
Unilateral/bilateral	Bilateral
Set up	Treadmill-based (support structure)
Exoskeleton weight	25 kg
Total DoFs	2 (Hip_FE, Knee_FE)
Actuated DoFs	2 (Hip_FE, Knee_FE)
Walking speed	N/A

Control

Control strategies	· Stiffness control<br>· Torque control

Electric power supply

Battery autonomy	N/A

Related publications

[1] Accoto D, Carpino G, Sergi F, Luigi N, Zollo L, Guglielmelli E. Design and Characterization of a Novel High-Power Series Elastic Actuator for a Lower Limb Robotic Orthosis. Int J Adv Robot Syst. 2013;10. doi:10.5772/56927.

[2] Sergi F, Accoto D, Tagliamonte NL, Carpino G, Guglielmelli E. A systematic graph-based method for the kinematic synthesis of non-anthropomorphic wearable robots for the lower limbs. Front Mech Eng. 2011;6:61–70. doi:10.1007/s11465-011-0206-2.

[3] Tagliamonte NL, Sergi F, Carpino G, Accoto D, Guglielmelli E. Human-Robot Interaction Tests on a Novel Robot for Gait Assistance. In: 2013 IEEE International Conference on Rehabilitation Robotics (ICORR). 2013. p. 1–6. doi:10.1109/ICORR.2013.6650387.

[4] Sergi F, Accoto D, Tagliamonte NL, Carpino G, Galzerano S, Guglielmelli E. Kinematic synthesis, optimization and analysis of a non-anthropomorphic 2-DOFs wearable orthosis for gait assistance. In: 2012 IEEE/RSJ International Conference on Intelligent Robots and Systems (IROS). Vilamoura (Portugal); 2012. doi:10.1109/IROS.2012.6386260.

Actuators

Type	SEA
Location	On-board
Power	N/A
Maximum torque	60 Nm
Maximum speed	N/A
Bandwidth	N/A
Weight	N/A
Modular actuators	✓
Actuator specific characteristics	
Spring typology	Inherent compliance of the Harmonic Drive
Spring stiffness	-
Actuator stiffness	24 kNm/rad

Structure & interface attachment components

Allowable wearer maximum weight	N/A
Allowable wearer height range	N/A
Anthropomorphic considerations	N/A
Configurable dimensions	· Hip axis-knee axis distance<br>· Knee axis-ankle axis distance
Structure typology	Rigid structure
Braces/segment	1

Applicability & general characteristics

Target user	Adults
Application	Assistance (SCI)
Unilateral/bilateral	Bilateral
Set up	Ambulatory (crutches)
Exoskeleton weight	N/A
Total DoFs	2 (Hip_FE, Knee_FE)
Actuated DoFs	2 (Hip_FE, Knee_FE)
Walking speed	0.20 m/s

Control

Control strategies	· Compliant control<br>· Position control<br>· Torque control

Electric power supply

Battery autonomy	N/A

Related publications

[1] Neuhaus PD, Noorden JH, Craig TJ, Torres T, Kirschbaum J, Pratt JE. Design and Evaluation of Mina a Robotic Orthosis for Paraplegics. In: 2011 IEEE International Conference on Rehabilitation Robotics (ICORR). Zurich (Switzerland); 2011. doi:10.1109/ICORR.2011.5975468.

[2] Raj AK, Neuhaus PD, Moucheboeuf AM, Noorden JH, Lecoutre D V. Mina: A Sensorimotor Robotic Orthosis for Mobility Assistance. J Robot. 2011;2011. doi:10.1155/2011/284352.

Actuators

Type	SEA
Location	On-board
Power	N/A
Maximum torque	N/A
Maximum speed	N/A
Bandwidth	N/A
Weight	1 kg
Modular actuators	-
Actuator specific characteristics	
Spring typology	Torsional spring
Spring stiffness	N/A
Actuator stiffness	N/A

Structure & interface attachment components

Allowable wearer maximum weight	N/A
Allowable wearer height range	N/A
Anthropomorphic considerations	N/A
Configurable dimensions	N/A
Structure typology	Rigid structure
Braces/segment	1

Applicability & general characteristics

Target user	Adults
Application	Assistance (Elderly)
Unilateral/bilateral	Bilateral
Set up	Ambulatory
Exoskeleton weight	8 kg
Total DoFs	1 (Hip_FE)
Actuated DoFs	1 (Hip_FE)
Walking speed	N/A

Control

Control strategies	Torque control

Electric power supply

Battery autonomy	N/A

Related publications

[1] Parri A, Yan T, Giovacchini F, Cortese M, Muscolo M, Fantozzi M, et al. A portable active pelvis orthosis for ambulatory movement assistance. In: Wearable robotics: challenges and trends; proceedings of the 2nd International Symposium on Wearable Robotics (WeRob2016). La Granja (Segovia), Spain; 2016. p. 75–80.

Actuators

Type	CompAct-ARS (VSA)
Location	On-board
Power	N/A
Maximum torque	80 Nm
Maximum speed	N/A
Bandwidth	N/A
Weight	N/A
Modular actuators	-
Actuator specific characteristics	
Spring typology	Linear spring
Spring stiffness	55 N/mm
Actuator stiffness	N/A

Structure & interface attachment components

Allowable wearer maximum weight	82.50 kg
Allowable wearer height range	N/A
Anthropomorphic considerations	N/A
Configurable dimensions	N/A
Structure typology	Rigid structure
Braces/segment	N/A

Applicability & general characteristics

Target user	Adults
Application	Assistance
Unilateral/bilateral	Bilateral
Set up	Ambulatory
Exoskeleton weight	N/A
Total DoFs	1 (Knee_FE)
Actuated DoFs	1 (Knee_FE)
Walking speed	N/A

Control

Control strategies	Assistive control

Electric power supply

Battery autonomy	N/A

Related publications

[1] Karavas NC, Tsagarakis NG, Saglia J, Galdwell DG. A Novel Actuator with Reconfigurable Stiffness for a Knee Exoskeleton : Design and Modeling. In: Springer, editor. Advances in Reconfigurable Mechanisms and Robots I. London (UK); 2012. p. 411–21. doi:10.1007/978-1-4471-4141-9.

[2] Karavas NC, Tsagarakis NG, Caldwell DG. Design, Modeling and Control of a Series Elastic Actuator for an Assistive Knee Exoskeleton. In: 4th IEEE RAS/EMBS International Conference on Biomedical Robotics and Biomechatronics (BioRob). Roma (Italy); 2012. p. 1813–9. doi:10.1109/BioRob.2012.6290757.

Actuators

Type	Pneumatic actuator – PAM
Location	On-board/off-board
Power	N/A
Maximum torque	N/A
Maximum speed	N/A
Bandwidth	N/A
Weight	N/A
Modular actuators	×

Actuator specific characteristics	
Pnumatic muscles/leg	4
Artificial muscle diameter/length	N/A / N/A
Artificial muscle maximum pressure	N/A
Artificial muscle stiffness	N/A
Actuator stiffness	N/A

Structure & interface attachment components

Allowable wearer maximum weight	N/A
Allowable wearer height range	N/A
Anthropomorphic considerations	Measurements of the Mexican population [*]
Configurable dimensions	· Pelvic width: 387.5 – 477.5 mm<br>· Leg length: 925.6 – 1105.6 mm
Structure typology	Rigid structure
Braces/segment	N/A

Applicability & general characteristics

Target user	Adults
Application	Assistance/Rehabilitation (PA)
Unilateral/bilateral	Bilateral
Set up	Ambulatory
Exoskeleton weight	N/A
Total DoFs	7 (Hip_AA, Hip_EE, Hip_FE, Knee_AA, Knee_FE, Ankle_PS, Ankle_DP)
Actuated DoFs	2 (Hip_FE, Knee_FE)
Walking speed	N/A

Control

Control strategies	· PD control<br>· PID control<br>· Admittance control

Electric power supply

Battery autonomy	N/A

Air source

Location	On-board/Off-board
Autonomy	N/A
Maximum pressure	N/A

Related publications

[1] Aguilar-Sierra H, Yu W, Salazar S, Lopez R. Design and control of hybrid actuation lower limb exoskeleton. Adv Mech Eng. 2015;7:1–13. doi:10.1177/1687814015590988.

[2] Aguilar-Sierra H, Lopez R, Yu W, Salazar S, Lozano R. A lower limb exoskeleton with hybrid actuation. In: 5th IEEE RAS & EMBS International Conference on Biomedical Robotics and Biomechatronics (BioRob). Sao Paulo (Brazil); 2014. p. 695–700. doi:10.1109/BIOROB.2014.6913859.

[*] Prado León LR, González Muñoz EL, and Avila Chaurand R, Dimensiones antropométricas de población latinoamericana. Universidad de Guadalajara, 2012.

Actuators

Type	Pneumatic actuator – PAM
Location	On-board
Power	N/A
Maximum torque	N/A
Maximum speed	N/A
Badwidth	N/A
Weight	N/A
Modular actuators	✗
Actuator specific characteristics	
Pnumatic muscles/leg	8
Artificial muscle diameter/length	30 mm / 350-600 mm
Artificial muscle maximum pressure	370 kPa
Artificial muscle stiffness	395N/m, 590N/m, 730N/m
Actuator stiffness	N/A

Structure & interface attachment components

Allowable wearer maximum weight	N/A
Allowable wearer height range	N/A
Anthropomorphic considerations	NASA 'typical average adult' dimension considerations
Configurable dimensions	· Leg length (+/- 80 mm)<br>· Custom molded to each subject braces
Structure typology	Rigid structure
Braces/segment	Combined solution (1 thigh, 2 shank)

Applicability & general characteristics

Target user	Adults
Application	Rehabilitation (SCI/stroke)
Unilateral/bilateral	Bilateral
Set up	Treadmill-based (support structure)
Exoskeleton weight	11 kg
Total DoFs	5 (Hip_AA, Hip_EE, Hip_FE, Knee_FE, Ankle_DP)
Actuated DoFs	4 (Hip_AA, Hip_FE, Knee_FE, Ankle_DP)
Walking speed	N/A

Control

Control strategies	· PID control

Electric power supply

Battery autonomy	N/A

Air source

Location	Off-board
Autonomy	N/A
Maximum pressure	N/A

Related publications

[1] Costa N, Caldwell DG. Control of a biomimetic “soft-actuated” 10DoF lower body exoskeleton. In: 1st IEEE/RAS-EMBS International Conference on Biomedical Robotics and Biomechatronics (BioRob). Pisa (Italy); 2006. p. 495–501. doi:10.1109/BIOROB.2006.1639137.

[2] Costa N, Bezdicek M, Brown M, Gray JO, Caldwell DG, Hutchins S. Joint motion control of a powered lower limb orthosis for rehabilitation. Int J Autom Comput. 2006;3:271–81. doi:10.1007/s11633-006-0271-x.

Actuators

Type	Pneumatic actuator – McKibben artificial muscle
Location	On-board
Power	N/A
Maximum torque	N/A
Maximum speed	N/A
Bandwidth	N/A
Weight	N/A
Modular actuators	✗
Actuator specific characteristics	
Pneumatic muscles per leg	8
Artificial muscle diameter/length	N/A / 200 mm
Artificial muscle maximum pressure	400 kPa
Artificial muscle stiffness	N/A
Actuator stiffness	N/A

Structure & interface attachment components

Allowable wearer maximum weight	79 kg
Allowable wearer height range	175 cm
Anthropomorphic considerations	Requirements for the system were determined from the 50th percentile male [*]
Configurable dimensions	N/A
Structure typology	Non-rigid structure (soft exoskeleton)
Structure weight	N/A
Braces/segment	Multiple

Applicability & general characteristics

Target user	Adults
Application	Rehabilitation (SCI/stroke)/ PA
Unilateral/bilateral	Bilateral
Set up	Ambulatory
Exoskeleton weight	9.12 kg
Total DoFs	5 (Hip_AA, Hip_EE, Hip_FE, Knee_FE, Ankle_DP)
Actuated DoFs	2 (Hip_FE, Knee_FE)
Walking speed	N/A

Control

Control strategies	Timing based control

Electric power supply

Battery autonomy	N/A

Air source

Location	On-board
Autonomy	1.45 min
Maximum pressure	30600 kPa

Related publications

[1] Wehner M, Quinlivan B, Aubin PM, Martinez-Villalpando E, Baumann M, Stirling L, et al. A lightweight soft exosuit for gait assistance. In: 2013 IEEE International Conference on Robotics and Automation (ICRA). 2013. p. 3362–9. doi:10.1109/ICRA.2013.6631046.

[*] Hallemans A, De Clercq D, Otten B, Aerts P. 3D joint dynamics of walking in toddlers. A cross-sectional study spanning the first rapid development phase of walking. Gait & Posture 2005, 22:107-118.

Actuators

Type	Pneumatic actuator – McKibben artificial muscle
Location	On-board
Power	N/A
Maximum torque	N/A
Maximum speed	N/A
Bandwidth	N/A
Weight	N/A
Modular actuators	✘
Actuator specific characteristics	
Pnumatic muscles/leg	6
Artificial muscle diameter/length	25.40 mm / 400, 550, 650 mm
Artificial muscle maximum pressure	100, 200, 300, 350 kPa
Artificial muscle stiffness	N/A
Actuator stiffness	N/A

Structure & interface attachment components

Allowable wearer maximum weight	N/A
Allowable wearer height range	N/A
Anthropomorphic considerations	N/A
Configurable dimensions	· Hip axis-knee axis distance<br>· Knee axis-ankle axis distance
Structure typology	Rigid structure
Braces/segment	Combined solution (1 thigh, 2 shank)

Applicability & general characteristics

Target user	Adults
Application	Rehabilitation (SCI/stroke)
Unilateral/bilateral	Bilateral
Set up	Treadmill-based (body-weight support)
Exoskeleton weight	N/A
Total DoFs	2 (Hip_FE, Knee_FE)
Actuated DoFs	2 (Hip_FE, Knee_FE)
Walking speed	0.70 m/s

Control

Control strategies	· Torque control<br>· Position control<br>· Assist-as-needed control

Electric power supply

Battery autonomy	N/A

Air source

Location	Off-board
Autonomy	N/A
Maximum pressure	5000 kPa

Related publications

[1] Dao Q. Design and Evaluation of the Lower-limb Robotic Orthosis for Gait Rehabilitation Actuated by Pneumatic Artificial Muscle. In: 2nd International Conference on Biomedical Signal and Image Processing. 2017. p. 85–9. doi:10.1145/3133793.3133810.
[2] Dao QT, Yamamoto SI. Assist-as-Needed Control of a Robotic Orthosis Actuated by Pneumatic Artificial Muscle for Gait Rehabilitation. Appl Sci. 2018;8. doi:10.1007/978-981-10-9038-7_117.
[3] Mat Dzahir MA, Nobutomo T, Yamamoto SI. Development of body weight support gait training system using pneumatic mckibben actuators -Control of Lower Extremity Orthosis-*. In: 35th Annual International Conference of the IEEE EMBS. Osaka (Japan); 2013. p. 6417–20. doi:10.1109/EMBC.2013.6611023.
[4] Yamamoto SI, Shibata Y, Imai S, Nobutomo T, Miyoshi T. Development of gait training system powered by pneumatic actuator like human musculoskeletal system. In: 2011 IEEE International Conference on Rehabilitation Robotics (ICORR). Zurich (Switzerland); 2011. p. 8–11. doi:10.1109/ICORR.2011.5975452.

Actuators

Type	Rigid actuator
Location	On-board
Power	N/A
Maximum torque	N/A
Maximum speed	N/A
Badwidth	N/A
Weight	N/A
Modular actuators	✓
Actuator specific characteristics	
Extra spring/spring stiffness value	✗

Structure & interface attachment components

Allowable wearer maximum weight	N/A
Allowable wearer height range	N/A
Anthropomorphic considerations	N/A
Configurable dimensions	N/A
Structure typology	Non-rigid structure (soft exoskeleton)
Structure weight	N/A
Braces/segment	Customfitted garment

Applicability & general characteristics

Target user	Adults
Application	Assistance/rehabilitation (SCI/stroke/elderly)
Unilateral/bilateral	Bilateral
Set up	Ambulatory
Exoskeleton weight	N/A
Total DoFs	3 (Hip_FE, Knee_FE, Ankle_DP)
Actuated DoFs	3 (Hip_FE, Knee_FE, Ankle_DP)
Walking speed	N/A

Control

Control strategies	Biomimetic control

Electric power supply

Battery autonomy	N/A

Related publications

[1] Graf ES, De Eyto A, Sposito M, Pauli C, O'Sullivan L, Bauer CM, et al. Basic functionality of a prototype wearable assistive soft exoskeleton for people with gait impairments - a case study. In: 11th ACM International Conference on PErvasive Technologies Related to Assistive Environments. 2018. p. 202–7. doi:10.1145/3197768.3197779.
[2] Ortiz J, Natali C Di, Caldwell DG. XoSoft - Iterative Design of a Modular Soft Lower Limb Exoskeleton. In: Wearable robotics: challenges and trends; proceedings of the 4th International Symposium on Wearable Robotics (WeRob2018). Pisa (Italy); 2018. p. 351–5.

Asbeck et al. [1]

Actuators

Type	Rigid actuator
Location	On-board/off-board
Power	N/A
Maximum torque	N/A
Maximum speed	1.59 m/s
Bandwidth	N/A
Weight	N/A
Modular actuators	✓
Actuator specific characteristics	
Extra spring/spring stiffness value	✓/ N/A

Sturcture & interface attachment components

Allowable wearer maximum weight	N/A
Allowable wearer height range	N/A
Anthropomorphic considerations	N/A
Configurable dimensions	N/A
Structure typology	Non-rigid structure (soft exoskeleton)
Structure weight	N/A
Braces/segment	1

Applicability & general characteristics

Target user	Adults
Application	Assistance (PA)
Unilateral/bilateral	Bilateral
Set up	Ambulatory
Exoskeleton weight	6.60 kg
Total DoFs	2 (Hip_FE, Ankle_PF)
Actuated DoFs	2 (Hip_FE, Ankle_PF)
Walking speed	1.79 m/s

Control

Control strategies	Force-based position control

Electric power supply

Battery autonomy	N/A

Related publications

[1] Asbeck AT, DeRossi SMM, Holt KG, Walsh CJ. A Biologically-Inspired Soft Exosuit for Walking Assistance. Int J Rob Res. 2015;34:744–62. doi:10.1177/0278364914562476.
[2] Panizzolo FA, Galiana I, Asbeck AT, Siviy C, Schmidt K, Holt KG, et al. A biologically-inspired multi-joint soft exosuit that can reduce the energy cost of loaded walking. J Neuroeng Rehabil. 2016;13. doi:10.1186/s12984-016-0150-9.
[3] Asbeck AT, Dyer RJ, Larusson AF, Walsh CJ. Biologically-inspired soft exosuit. In: 2013 IEEE International Conference on Rehabilitation Robotics (ICORR). Seattle (USA); 2013. p. 24–6. doi:10.1109/ICORR.2013.6650455.
[4] Ding Y, Galiana I, Asbeck AT, De Rossi SMM, Bae J, Santos TRT, et al. Biomechanical and physiological evaluation of multi-joint assistance with soft exosuits. IEEE Trans Neural Syst Rehabil Eng. 2017;25:119–30. doi:10.1109/TNSRE.2016.2523250.
[5] Asbeck AT, Schmidt K, Galiana I, Wagner D, Walsh CJ. Multi-joint Soft Exosuit for Gait Assistance. In: 2015 IEEE International Conference on Robotics and Automation (ICRA). Seattle (USA); 2015. p. 6197–204. doi:10.13140/RG.2.1.2874.5687.

Quinlivan et al. [1]

Actuators

Type	Rigid actuator
Location	Off-board
Power	N/A
Maximum torque	N/A
Maximum speed	N/A
Bandwidth	N/A
Weight	N/A
Modular actuators	✗
Actuator specific characteristics	
Extra spring/spring stiffness value	✗

Structure & interface attachment components

Allowable wearer maximum weight	N/A
Allowable wearer height range	N/A
Anthropomorphic considerations	N/A
Configurable dimensions	N/A
Structure typology	Non-rigid structure (soft exoskeleton)
Structure weight	0.89 kg
Braces/segment	1

Applicability & general characteristics

Target user	Adults
Application	Assistance
Unilateral/bilateral	Bilateral
Set up	Ambulatory
Exoskeleton weight	N/A
Total DoFs	2 (Hip_F, Ankle_P)
Actuated DoFs	2 (Hip_F, Ankle_P)
Walking speed	1.50 m/s

Control

Control strategies	Force-based position control

Electric power supply

Battery autonomy	N/A

Related publications

[1] Quinlivan BT, Lee S, Malcolm P, Rossi DM, Grimmer M, Siviy C, et al. Assistance magnitude versus metabolic cost reductions for a tethered multiarticular soft exosuit. Sci Robot. 2017;2. doi:10.1126/scirobotics.aah4416.

Ding et al. [1]

Actuators

Type	Rigid actuator
Location	Off-board
Power	N/A
Maximum torque	N/A
Maximum speed	3.20 m/s
Badwidth	19.60 Hz
Weight	N/A
Modular actuators	✗
Actuator specific characteristics	
Extra spring/spring stiffness value	✗

Structure & interface attachment components

Allowable wearer maximum weight	N/A
Allowable wearer height range	N/A
Anthropomorphic considerations	N/A.
Configurable dimensions	N/A
Structure typology	Non-rigid structure (soft exoskeleton)
Structure weight	0.86 kg
Braces/segment	1

Applicability & general characteristics

Target user	Adults
Application	Assistance
Unilateral/bilateral	Bilateral
Set up	Ambulatory
Exoskeleton weight	N/A
Total DoFs	1 (Hip_E)
Actuated DoFs	1 (Hip_E)
Walking speed	1.25 m/s

Control

Control strategies	· Admittance-force control<br>· Admittance control<br>· Position control

Electric power supply

Battery autonomy	N/A

Related publications

[1] Ding Y, Kim M, Kuindersma S, Walsh CJ. Human-in-the-loop optimization of hip assistance with a soft exosuit during walking. Sci Robot. 2018;3. doi:10.1111/irfi.12020.
[2] Lee G, Ding Y, Bujanda IG, Karavas N, Zhou YM, Walsh CJ. Improved assistive profile tracking of soft exosuits for walking and jogging with off-board actuation. In: 2017 IEEE/RSJ International Conference on Intelligent Robots and Systems (IROS). Vancouver (Canada); 2017. p. 1699–706. doi:10.1109/IROS.2017.8205981.
[3] Ding Y, Panizzolo FA, Siviy C, Malcolm P, Galiana I, Holt KG, et al. Effect of timing of hip extension assistance during loaded walking with a soft exosuit. J Neuroeng Rehabil. 2016;13:87. doi:10.1186/s12984-016-0196-8.
[4] Ding Y, Galiana I, Siviy C, Panizzolo FA, Walsh C. IMU-based iterative control for hip extension assistance with a soft exosuit. In: 2016 IEEE International Conference on Robotics and Automation (ICRA). Stockholm (Sweden); 2016. p. 3501–8. doi:10.1109/ICRA.2016.7487530.

Huang et al. [1]

Actuators

Type	SEA
Location	On-board
Power	N/A
Maximum torque	80 Nm
Maximum speed	5 rad/s
Bandwidth	N/A
Weight	N/A
Modular actuators	✓
Actuator specific characteristics	
Spring typology	Custom made disc-shaped torsional spring
Spring stiffness	N/A
Actuator stiffness	N/A

Structure & interface attachment components

Allowable wearer maximum weight	N/A
Allowable wearer height range	N/A
Anthropomorphic considerations	N/A
Configurable dimensions	· Hip axis-knee axis distance<br>· Knee axis-ankle axis distance
Structure typology	Rigid structure
Braces/segment	2

Applicability & general characteristics

Target user	Adults
Application	Rehabilitation
Unilateral/bilateral	Unilateral
Set up	Ambulatory
Exoskeleton weight	N/A
Total DoFs	3 (Hip_FE, Knee_FE, Ankle_DP)
Actuated DoFs	2 (Hip_FE, Knee_FE)
Walking speed	N/A

Control

Control strategies	N/A

Electric power supply

Battery autonomy	N/A

Related publications

[1] Huang C, Chen W, Liu J, Zhang J. Design of a compliant joint actuator for lower-limb exoskeleton robot. In: 12th IEEE Conference on Industrial Electronics and Applications (ICIEA). 2017. p. 1522–7. doi:10.1109/ICIEA.2017.8283080.

Actuators

Type	SEA
Location	On-board
Power	N/A
Maximum torque	120 Nm
Maximum speed	0.26 m/s
Bandwidth	2600 N-5 Hz; 900 N-17 Hz
Weight	1.38 kg
Modular actuators	✗
Actuator specific characteristics	
Spring typology	Linear spring
Spring stiffness	375-435 N/mm
Actuator stiffness	N/A

Structure & interface attachment components

Allowable wearer maximum weight	75 kg
Allowable wearer height range	N/A
Anthropomorphic considerations	N/A
Configurable dimensions	N/A
Structure typology	Rigid structure
Braces/segment	2

Applicability & general characteristics

Target user	Adults
Application	Rehabilitation/ PA
Unilateral/bilateral	Unilateral
Set up	Ambulatory
Exoskeleton weight	3.86 kg
Total DoFs	3 (Knee_FE, Ankle_DP, Ankle_PS)
Actuated DoFs	2 (Knee_FE, Ankle_DP)
Walking speed	N/A

Control

Control strategies	Force control

Electric power supply

Battery autonomy	N/A

Related publications

[1] Beil J, Perner G, Asfour T. Design and Control of the Lower Limb Exoskeleton KIT-EXO-1. In: 2015 IEEE International Conference on Rehabilitation Robotics (ICORR). Singapore (Singapore); 2015. p. 119–24. doi:10.1109/ICORR.2015.7281186.

Zhang et al. [1]

Actuators

Type	SEA
Location	On-board
Power	N/A
Maximum torque	N/A
Maximum speed	N/A
Bandwidth	N/A
Weight	N/A
Modular actuators	✘
Actuator specific characteristics	
Spring typology	Linear spring, Bowden cable
Spring stiffness	17.97 N/mm
Actuator stiffness	N/A

Structure & interface attachment components

Allowable wearer maximum weight	70 kg
Allowable wearer height range	N/A
Anthropomorphic considerations	“GB 10000-1988 Chinese adults body size” issued by the China National Standardization Management Committee
Configurable dimensions	N/A
Structure typology	Rigid structure
Braces/segment	1

Applicability & general characteristics

Target user	Adults
Application	Rehabilitation
Unilateral/bilateral	Unilateral
Set up	Ambulatory
Exoskeleton weight	N/A
Total DoFs	3 (Ankle_PS, Ankle_EE, Ankle_DP)
Actuated DoFs	1 (Ankle_DP)
Walking speed	1 m/s

Control

Control strategies	N/A

Electric power supply

Battery autonomy	N/A

Related publications

[1] Zhang C, Zhu Y, Fan J, Zhao J, Yu H. Design of a quasi-passive 3 DOFs ankle-foot wearable rehabilitation orthosis. Biomed Mater Eng. 2015;26:S647–54. doi:10.3233/BME-151356.

Actuators

Type	SEA (*=knee actuator, **=ankle actuator)
Location	On-board
Power	N/A
Maximum torque	N/A
Maximum speed	N/A
Bandwidth	N/A
Weight	N/A
Modular actuators	✗
Actuator specific characteristics	
Spring typology	Linear spring
Spring stiffness	0.17 N/mm*, 0.18 N/mm**
Actuator stiffness	N/A

Structure & interface attachment components

Allowable wearer maximum weight	75 kg
Allowable wearer height range	N/A
Anthropomorphic considerations	N/A
Configurable dimensions	N/A
Structure typology	Rigid structure
Braces/segment	1

Applicability & general characteristics

Target user	Adults
Application	Rehabilitation
Unilateral/bilateral	Unilateral
Set up	Ambulatory
Exoskeleton weight	N/A
Total DoFs	2 (Knee_FE, Ankle_DP)
Actuated DoFs	2 (Knee_FE, Ankle_DP)
Walking speed	N/A

Control

Control strategies	N/A

Electric power supply

Battery autonomy	N/A

Related publications

[1] Lopez R, Aguilar-Sierra H, Salazar S, Lozano R. Model and Control of the ELLTIO with two degrees of freedom. In: 17th International Conference on System Theory, Control and Computing (ICSTCC). 2013. p. 305–10. doi:10.1109/ICSTCC.2013.6688977.

[2] Lopez R, Salazar S, Torres J, Lozano R. Modeling and Control of a Lower Limb Exoskeleton with two degrees of freedom. In: 9th International Conference on Electrical Engineering, Computing Science and Automatic Control (CEE). Mexico City (Mexico); 2012. doi:10.1109/ICEEE.2012.6421205.

Actuators

Type	SEA
Location	On-board
Power	120 W (motor)
Maximum torque	60 Nm
Maximum speed	N/A
Bandwidth	16 Hz; 64 Hz
Weight	0.85 kg
Modular actuators	✓
Actuator specific characteristics	
Spring typology	Linear spring, torsional spring
Spring stiffness	24 N/mm; 0.29 Nm/rad
Actuator stiffness	N/A

Structure & interface attachment components

Allowable wearer maximum weight	70 kg
Allowable wearer height range	N/A
Anthropomorphic considerations	N/A
Configurable dimensions	N/A
Structure typology	Rigid structure
Braces/segment	1

Applicability & general characteristics

Target user	Adults
Application	Rehabilitation (Stroke)
Unilateral/bilateral	Unilateral
Set up	Ambulatory
Exoskeleton weight	3.50 kg
Total DoFs	2 (Knee_FE, Ankle_DP)
Actuated DoFs	2 (Knee_FE, Ankle_DP)
Walking speed	N/A

Control

Control strategies	· Assistive force control<br>· Force control<br>· Human-in-charge control

Electric power supply

Battery autonomy	N/A

Related publications

[1] Yu H, Huang S, Chen G, Pan Y, Guo Z. Human – Robot Interaction Control of Rehabilitation Robots With Series Elastic Actuators. IEEE Trans Robot. 2015;31:1089–100. doi:10.1109/TRO.2015.2457314.

[2] Mizanoor Rahman SM. Design of a Modular Knee-Ankle-Foot-Orthosis Using Soft Actuator for Gait Rehabilitation. In: 14th Annual Conference Towards Autonomous Robotic Systems. 2013. p. 195–209. doi:10.1007/978-3-662-43645-5.

[3] Chen G, Yu H, Cruz MSTA, Chen G, Huang S, Zhu C, et al. Mechanical design of a portable knee-ankle-foot robot. In: 2013 IEEE International Conference on Robotics and Automation (ICRA). Karlsruhe (Germany); 2013. doi:10.1109/ICRA.2013.6630870.

[4] Chen G, Qi P, Guo Z, Yu H. Mechanical design and evaluation of a compact portable knee – ankle – foot robot for gait rehabilitation. Mech Mach Theory. 2016;103:51–64. doi:10.1016/j.mechmachtheory.2016.04.012.

Actuators

Type	SEA
Location	On-board
Power	N/A
Maximum torque	6 Nm
Maximum speed	N/A
Bandwidth	N/A
Weight	N/A
Modular actuators	✓
Actuator specific characteristics	
Spring typology	Torsional spring
Spring stiffness	3 Nm/rad
Actuator stiffness	N/A

Structure & interface attachment components

Allowable wearer maximum weight	24.50 kg
Allowable wearer height range	N/A
Anthropomorphic considerations	N/A
Configurable dimensions	The frame length is adjustable.
Structure typology	Rigid structure
Braces/segment	1

Applicability & general characteristics

Target user	Children
Application	Rehabilitation (CP)
Unilateral/bilateral	Unilateral
Set up	Ambulatory
Exoskeleton weight	2.50 kg
Total DoFs	2 (Knee_FE, Ankle_DP)
Actuated DoFs	2 (Knee_FE, Ankle_DP)
Walking speed	N/A

Control

Control strategies	· Force control<br>· Spring deflection control

Electric power supply

Battery autonomy	N/A

Related publications

[1] Rossi S, Patanè F, Sette F Del, Cappa P. WAKE-up: a Wearable Ankle Knee Exoskeleton. In: 5th IEEE RAS & EMBS International Conference on Biomedical Robotics and Biomechatronics (BioRob). Sao Paulo (Brazil); 2014. p. 504–7. doi:10.1109/BIOROB.2014.6913827.

Actuators

Type	SEA
Location	On-board
Power	N/A
Maximum torque	780 Nm
Maximum speed	6.81 rad/s
Bandwidth	N/A
Weight	N/A
Modular actuators	-
Actuator specific characteristics	
Spring typology	Linear spring, Bowden cable
Spring stiffness	10 N/mm
Actuator stiffness	26 Nm/rad

Structure & interface attachment components

Allowable wearer maximum weight	N/A
Allowable wearer height range	N/A
Anthropomorphic considerations	N/A
Configurable dimensions	N/A
Structure typology	Rigid structure
Braces/segment	1

Applicability & general characteristics

Target user	Adults
Application	Rehabilitation (stroke)
Unilateral/bilateral	Unilateral
Set up	Ambulatory
Exoskeleton weight	N/A
Total DoFs	1 (Knee_FE)
Actuated DoFs	1 (Knee_FE)
Walking speed	N/A

Control

Control strategies	Force control

Electric power supply

Battery autonomy	N/A

Related publications

[1] Celebi B, Yalcin M, Patoglu V. ASSISTON-KNEE: A Self-Aligning Knee Exoskeleton. In: 2013 IEEE/RSJ International Conference on Intelligent Robots and Systems (IROS). Tokyo (Japan); 2013. p. 996–1002. doi:10.1109/IROS.2013.6696472.

Choi et al. [1]

Actuators

Type	C-DSSAS (SEA)
Location	On-board
Power	N/A
Maximum torque	N/A
Maximum speed	N/A
Bandwidth	N/A
Weight	N/A
Modular actuators	-
Actuator specific characteristics	
Spring typology	Spiral spring
Spring stiffness	6.60 Nm/rad, 8.25 Nm/rad, 11.02 Nm/rad
Actuator stiffness	N/A

Structure & interface attachment components

Allowable wearer maximum weight	N/A
Allowable wearer height range	N/A
Anthropomorphic considerations	N/A
Configurable dimensions	N/A
Structure typology	Rigid structure
Braces/segment	2

Applicability & general characteristics

Target user	Adults
Application	Rehabilitation
Unilateral/bilateral	Unilateral
Set up	Ambulatory
Exoskeleton weight	N/A
Total DoFs	1 (Knee_FE)
Actuated DoFs	1 (Knee_FE)
Walking speed	N/A

Control

Control strategies	Torque control

Electric power supply

Battery autonomy	N/A

Related publications

[1] Choi W, Won J, Lee J, Park J. Low stiffness design and hysteresis compensation torque control of SEA for active exercise rehabilitation robots. Auton Robots. 2017;41:1221–42. doi:10.1007/s10514-016-9591-z.

Actuators

Type	SEA
Location	On-board
Power	N/A
Maximum torque	15 Nm
Maximum speed	5.76 rad/s
Bandwidth	5 Nm-9.6 Hz
Weight	2.53 kg
Modular actuators	-
Actuator specific characteristics	
Spring typology	Custom made disc-shaped torsional spring
Spring stiffness	200 Nm/rad
Actuator stiffness	N/A

Structure & interface attachment components

Allowable wearer maximum weight	70 kg
Allowable wearer height range	N/A
Anthropomorphic considerations	N/A
Configurable dimensions	N/A
Structure typology	Rigid structure
Braces/segment	N/A

Applicability & general characteristics

Target user	Adults
Application	Rehabilitation
Unilateral/bilateral	Bilateral
Set up	Ambulatory
Exoskeleton weight	N/A
Total DoFs	1 (Knee_FE)
Actuated DoFs	1 (Knee_FE)
Walking speed	N/A

Control

Control strategies	· Impedance control<br>· Troque control

Electric power supply

Battery autonomy	N/A

Related publications

[1] dos Santos WM, Caurin GAP, Siqueira AAG. Design and control of an active knee orthosis driven by a rotary Series Elastic Actuator. Control Eng Pract. 2017;58:307–18. doi:10.1016/j.conengprac.2015.09.008.
[2] dos Santos WM, Siqueira AAG. Impedance Control of a Rotary Series Elastic Actuator for Knee Rehabilitation. In: 19th World Congress The International Federation of Automatic Control. Cape Town (South Africa): IFAC; 2014. p. 4801–6. doi:10.3182/20140824-6-ZA-1003.00987.
[3] dos Santos WM, Caurin GAP, Siqueira AAG. Torque Control Characterization of a Rotary Series Elastic Actuator for Knee Rehabilitation. In: 16th International Conference on Advanced Robotics (ICAR). Montevideo (Uruguay); 2013. doi:10.1109/ICAR.2013.6766567.

Actuators

Type	SEA
Location	On-board
Power	N/A
Maximum torque	N/A
Maximum speed	N/A
Bandwidth	N/A
Weight	N/A
Modular actuators	-
Actuator specific characteristics	
Spring typology	Linear spring, Bowden cable
Spring stiffness	32 N/mm
Actuator stiffness	N/A

Structure & interface attachment components

Allowable wearer maximum weight	N/A
Allowable wearer height range	N/A
Anthropomorphic considerations	N/A
Configurable dimensions	N/A
Structure typology	Rigid structure
Braces/segment	1

Applicability & general characteristics

Target user	Adults
Application	Rehabilitation
Unilateral/bilateral	Unilateral
Set up	Non-ambulatory
Exoskeleton weight	N/A
Total DoFs	1 (Knee_FE)
Actuated DoFs	1 (Knee_FE)
Walking speed	N/A

Control

Control strategies	N/A

Electric power supply

Battery autonomy	N/A

Related publications

[1] Ren Y, Member S, Zhang D, Member S. FEXO Knee : A Rehabilitation Device for Knee Joint Combining Functional Electrical Stimulation with a Compliant Exoskeleton. In: 5th IEEE RAS & EMBS International Conference on Biomedical Robotics and Biomechatronics (BioRob). Sao Paulo (Brazil); 2014. p. 683–8. doi:10.1109/BIOROB.2014.6913857.

Actuators

Type	SEA
Location	On-board
Power	N/A
Maximum torque	140 Nm
Maximum speed	N/A
Bandwidth	N/A
Weight	N/A
Modular actuators	-
Actuator specific characteristics	
Spring typology	Torsional spring
Spring stiffness	N/A
Actuator stiffness	1000 Nm/rad

Structure & interface attachment components

Allowable wearer maximum weight	N/A
Allowable wearer height range	N/A
Anthropomorphic considerations	N/A
Configurable dimensions	N/A
Structure typology	Rigid structure
Braces/segment	Combined solution (2 thigh, 1 shank)

Applicability & general characteristics

Target user	Adults
Application	Assistance/PA
Unilateral/bilateral	Unilateral
Set up	Ambulatory
Exoskeleton weight	3.75 kg
Total DoFs	6 (Knee_FE, other knee joint DoFs)
Actuated DoFs	1 (Knee_FE)
Walking speed	1.37 m/s

Control

Control strategies	Torque control

Electric power supply

Battery autonomy	N/A

Related publications

[1] Saccares L, Sarakoglou I, Tsagarakis NG. iT-Knee: An exoskeleton with ideal torque transmission interface for ergonomic power augmentation. In: 2016 IEEE/RSJ International Conference on Intelligent Robots and Systems (IROS). Daejeon (Korea); 2016. p. 780–6. doi:10.1109/IROS.2016.7759140.

Kong et al. [1]

Actuators

Type	cRSEA
Location	On-board
Power	N/A
Maximum torque	130 Nm
Maximum speed	N/A
Bandwidth	20 Hz
Weight	N/A
Modular actuators	-
Actuator specific characteristics	
Spring typology	Torsional spring
Spring stiffness	0.30 Nm/rad
Actuator stiffness	N/A

Structure & interface attachment components

Allowable wearer maximum weight	70 kg
Allowable wearer height range	N/A
Anthropomorphic considerations	N/A
Configurable dimensions	N/A
Structure typology	Rigid structure
Braces/segment	1

Applicability & general characteristics

Target user	Adults
Application	Assistance
Unilateral/bilateral	Unilateral
Set up	Ambulatory
Exoskeleton weight	N/A
Total DoFs	1 (Knee_FE)
Actuated DoFs	1 (Knee_FE)
Walking speed	N/A

Control

Control strategies	Torque control

Electric power supply

Battery autonomy	N/A

Related publications

[1] Kong K, Bae J, Tomizuka M. A compact rotary series elastic actuator for human assistive systems. IEEE/ASME Trans Mechatronics. 2012;17:288–97. doi:10.1109/TMECH.2010.2100046.

Actuators

Type	SEA
Location	On-board
Power	N/A
Maximum torque	71 Nm
Maximum speed	N/A
Bandwidth	N/A
Weight	N/A
Modular actuators	×
Actuator specific characteristics	
Spring typology	Linear spring
Spring stiffness	N/A
Actuator stiffness	N/A

Structure & interface attachment components

Allowable wearer maximum weight	N/A
Allowable wearer height range	155 – 180 cm
Anthropomorphic considerations	N/A
Configurable dimensions	Braces longitudinal position
Structure typology	Rigid structure
Braces/segment	1

Applicability & general characteristics

Target user	Adults
Application	Assistance
Unilateral/bilateral	Unilateral
Set up	Ambulatory
Exoskeleton weight	N/A
Total DoFs	1 (Knee_FE)
Actuated DoFs	1 (Knee_FE)
Walking speed	N/A

Control

Control strategies	Force control

Electric power supply

Battery autonomy	N/A

Related publications

[1] Luo Y, Wang C, Wang Z, Ma Y, Wang C, Wu X. Design and control for a compliant knee exoskeleton. In: 2017 IEEE International Conference on Information and Automation (ICIA). Macau (China); 2017. p. 282–7. doi:10.1109/ICInfA.2017.8078920.

Actuators

Type	SEA
Location	On-board
Power	634 W (actuator)
Maximum torque	N/A
Maximum speed	0.28 rad/s
Bandwidth	7.5 Hz; 35 Hz
Weight	1.13 kg
Modular actuators	✘
Actuator specific characteristics	
Spring typology	Linear spring
Spring stiffness	N/A
Actuator stiffness	N/A

Structure & interface attachment components

Allowable wearer maximum weight	60 kg
Allowable wearer height range	N/A
Anthropomorphic considerations	N/A.
Configurable dimensions	N/A
Structure typology	Rigid structure
Braces/segment	1

Applicability & general characteristics

Target user	Adults
Application	Assistance (PA)
Unilateral/bilateral	Unilateral
Set up	Ambulatory
Exoskeleton weight	3.00 kg
Total DoFs	1 (Knee_FE)
Actuated DoFs	1 (Knee_FE)
Walking speed	2.50 m/s

Control

Control strategies	Force control

Electric power supply

Battery autonomy	N/A

Related publications

[1] Pratt JE, Krupp BT, Morse CJ, Collins SH. The RoboKnee: an exoskeleton for enhancing strength and endurance during walking. In: 2004 IEEE International Conference on Robotics and Automation (ICRA). New Orleans (USA); 2004. p. 2430–5. doi:10.1109/ROBOT.2004.1307425.

Sulzer et al. [1]

Actuators

Type	SEA
Location	On-board
Power	634 W
Maximum torque	41 Nm
Maximum speed	N/A
Bandwidth	4 Hz; 8 Hz
Weight	N/A
Modular actuators	✗
Actuator specific characteristics	
Spring typology	Torsional spring, Bowden cable
Spring stiffness	24 Nm/rad
Actuator stiffness	N/A

Structure & interface attachment components

Allowable wearer maximum weight	N/A
Allowable wearer height range	N/A
Anthropomorphic considerations	N/A
Configurable dimensions	N/A
Structure typology	Rigid structure
Braces/segment	1

Applicability & general characteristics

Target user	Adults
Application	Assistance (Stroke)
Unilateral/bilateral	Unilateral
Set up	Treadmill-based (body-weight support structure)
Exoskeleton weight	1.20 kg
Total DoFs	1 (Knee_FE)
Actuated DoFs	1 (Knee_FE)
Walking speed	1.10 m/s

Control

Control strategies	Torque control

Electric power supply

Battery autonomy	N/A

Related publications

[1] Sulzer JS, Roiz RA, Peshkin MA, Patton JL. A Highly Backdrivable, Lightweight Knee Actuator for Investigating Gait in Stroke. IEEE Trans Robot. 2009;25:539–48. doi:10.1109/TRO.2009.2019788.A.

Actuators

Type	SEA
Location	On-board
Power	N/A
Maximum torque	N/A
Maximum speed	1 m/s
Bandwidth	16 Hz; 24 Hz
Weight	0.95 kg
Modular actuators	✓
Actuator specific characteristics	
Spring typology	Linear spring, Bowden cable
Spring stiffness	10 N/mm
Actuator stiffness	N/A

Structure & interface attachment components

Allowable wearer maximum weight	N/A
Allowable wearer height range	N/A
Anthropomorphic considerations	N/A
Configurable dimensions	N/A
Structure typology	Rigid structure
Braces/segment	1

Applicability & general characteristics

Target user	Adults
Application	Rehabilitation (stroke)
Unilateral/bilateral	Unilateral
Set up	Ambulatory
Exoskeleton weight	4.25 kg
Total DoFs	3 (Ankle_DP, Ankle_EE, Ankle_PS)
Actuated DoFs	2 (Ankle_DP, Ankle_PS)
Walking speed	N/A

Control

Control strategies	· Force control<br>· Zero impedance control

Electric power supply

Battery autonomy	N/A

Related publications

[1] Erdogan A, Celebi B, Satici AC, Patoglu V. Assist On-Ankle: a reconfigurable ankle exoskeleton with series-elastic actuation. Auton Robots. 2017;41:743–58. doi:10.1007/s10514-016-9551-7.

Actuators

Type	SEA
Location	On-board
Power	77 W
Maximum torque	N/A
Maximum speed	N/A
Bandwidth	N/A
Weight	0.50 kg
Modular actuators	-
Actuator specific characteristics	
Spring typology	Linear spring
Spring stiffness	N/A
Actuator stiffness	N/A

Structure & interface attachment components

Allowable wearer maximum weight	80 kg
Allowable wearer height range	N/A
Anthropomorphic considerations	N/A
Configurable dimensions	N/A
Structure typology	Rigid structure
Braces/segment	1

Applicability & general characteristics

Target user	Adults
Application	Assistance
Unilateral/bilateral	Unilateral
Set up	Ambulatory
Exoskeleton weight	1.75 kg
Total DoFs	1 (Ankle_DP)
Actuated DoFs	1 (Ankle_DP)
Walking speed	1.25 m/s

Control

Control strategies	· Position control<br>· Stiffness control<br>· Velocity control

Electric power supply

Battery autonomy	N/A

Related publications

[1] Boehler AW, Hollander KW, Sugar TG, Shin D. Design, Implementation and Test Results of a Robust Control Method for a Powered Ankle Foot Orthosis (AFO). In: 2008 IEEE International Conference on Robotics and Automation (ICRA). Pasadena (USA); 2008. p. 2025–30. doi:10.1109/ROBOT.2008.4543504.

[2] Hollander KW, Ilg R, Sugar TG, Herring D. An Efficient Robotic Tendon for Gait Assistance. J Biomech Eng. 2006;128:788–91. doi:10.1115/1.2264391.

[3] Hitt J, Oymagil AM, Sugar T, Hollander K, Boehler A, Fleeger J. Dynamically controlled ankle-foot orthosis (DCO) with regenerative kinetics: Incrementally attaining user portability. In: 2007 IEEE International Conference on Robotics and Automation (ICRA). Roma (Italy); 2007. p. 1541–6. doi:10.1109/ROBOT.2007.363543.

Jardim et al. [1]

Actuators

Type	SEA
Location	On-board
Power	N/A
Maximum torque	N/A
Maximum speed	N/A
Bandwidth	N/A
Weight	N/A
Modular actuators	-
Actuator specific characteristics	
Spring typology	Linear spring
Spring stiffness	78.90 N/mm
Actuator stiffness	N/A

Strucutre & interface attachment components

Allowable wearer maximum weight	N/A
Allowable wearer height range	N/A
Anthropomorphic considerations	Anthropometric measures of a healthy human were considered.
Configurable dimensions	N/A
Structure typology	Rigid structure
Braces/segment	1

Applicability & general characteristics

Target user	Adults
Application	Rehabilitation
Unilateral/bilateral	Unilateral
Set up	Ambulatory
Exoskeleton weight	N/A
Total DoFs	1 (Ankle_DP)
Actuated DoFs	1 (Ankle_DP)
Walking speed	N/A

Control

Control strategies	· Force control<br>· Impedance control<br>· Variable impedance control

Electric power supply

Battery autonomy	N/A

Related publications

[1] Jardim B, Siqueira AAG. Development and analysis of series elastic actuators for impedance control of an active ankle–foot orthosis. J Brazilian Soc Mech Sci Eng. 2014;36:501–10. doi:10.1007/s40430-013-0092-0.

Blaya et al. [1]

Actuators

Type	SEA
Location	On-board
Power	N/A
Maximum torque	N/A
Maximum speed	N/A
Bandwidth	N/A
Weight	N/A
Modular actuators	-
Actuator specific characteristics	
Spring typology	Linear spring
Spring stiffness	N/A
Actuator stiffness	N/A

Structure & interface attachment components

Allowable wearer maximum weight	N/A
Allowable wearer height range	N/A
Anthropomorphic considerations	N/A
Configurable dimensions	N/A
Structure typology	Rigid structure
Braces/segment	1

Applicability & general characteristics

Target user	Adults
Application	Rehabilitation
Unilateral/bilateral	Unilateral
Set up	Ambulatory
Exoskeleton weight	2.60 kg
Total DoFs	1 (Ankle_DP)
Actuated DoFs	1 (Ankle_DP)
Walking speed	N/A

Control

Control strategies	Impedance control

Electric power supply

Battery autonomy	N/A

Related publications

[1] Blaya JA, Herr H. Adaptive Control of a Variable-Impedance Ankle-Foot Orthosis to Assist Drop-Foot Gait. IEEE Trans Neural Syst Rehabil Eng. 2004;12:24–31. doi:10.1109/TNSRE.2003.823266.

Actuators

Type	SEA
Location	Off-board
Power	N/A
Maximum torque	N/A
Maximum speed	5.30 rad/s
Bandwidth	17.70 Hz – 50 Nm; 24.20 Hz – 20 Nm
Weight	N/A
Modular actuators	✗
Actuator specific characteristics	
Spring typology	Linear spring
Spring stiffness	15.10 N/mm, 27.50 N/mm, 50.10 N/mm, 103.10 N/mm, 235.7 N/mm
Actuator stiffness	108.90 Nm/rad, 160.40 Nm/rad, 212 Nm/rad, 269.30 Nm/rad, 320.90 Nm/rad, 228 Nm/rad

Structure & interface attachment components

Allowable wearer maximum weight	N/A
Allowable wearer height range	N/A
Anthropomorphic considerations	· User shank lengths ranging from 0.42 to 0.50 m<br>· Shoe sizes ranging from a women's size 7 to a men's size 12 (US)
Configurable dimensions	Shank length: + 0.04 m
Structure typology	Rigid structure
Braces/segment	1

Applicability & general characteristics

Target user	Adults
Application	Rehabilitation/assistance
Unilateral/bilateral	Unilateral
Set up	Ambulatory
Exoskeleton weight	0.87 kg
Total DoFs	1 (Ankle_P)
Actuated DoFs	1 (Ankle_P)
Walking speed	1.25 m/s

Control

Control strategies	· Proportional control<br>· Torque control

Electric power supply

Battery autonomy	N/A

Related publications

[1] Witte KA, Zhang J, Jackson RW, Collins SH. Design of two lightweight, high-bandwidth torque-controlled ankle exoskeletons. In: 2015 IEEE International Conference on Robotics and Automation (ICRA). Seattle (USA); 2015. p. 1223–8. doi:10.1109/ICRA.2015.7139347.

Actuators

Type	Pneumatic actuator – McKibben artificial muscle
Location	On-board
Power	N/A
Maximum torque	N/A
Maximum speed	N/A
Bandwidth	N/A
Weight	N/A
Modular actuators	✓
Actuator specific characteristics	
Pneumatic muscles per leg	4
Artificial muscle diameter/length	N/A / N/A
Artificial muscle maximum pressure	N/A
Artificial muscle stiffness	N/A
Actuator stiffness	N/A

Structure & interface attachment components

Allowable wearer maximum weight	N/A
Allowable wearer height range	N/A
Anthropomorphic considerations	N/A
Configurable dimensions	N/A
Structure typology	Non-rigid structure (soft exoskeleton)
Structure weight	N/A
Braces/segment	1

Applicability & general characteristics

Target user	Adults
Application	Assistance/Rehabilitation (Stroke/CP/MS)
Unilateral/bilateral	Unilateral
Set up	Ambulatory
Exoskeleton weight	0.95 kg
Total DoFs	2 (Ankle_PS, Ankle_DP)
Actuated DoFs	2 (Ankle_PS, Ankle_DP)
Walking speed	N/A

Control

Control strategies	PWM control

Electric power supply

Battery autonomy	2 hours

Air source

Location	Off-board
Autonomy	320 cycles
Maximum pressure	827 kPa

Related publications

[1] Park Y, Chen B, Young D, Stirling L, Wood RJ, Goldfield E, et al. Bio-inspired Active Soft Orthotic Device for Ankle Foot Pathologies. In: 2011 IEEE/RSJ International Conference on Intelligent Robots and Systems (IROS). San Francisco (USA); 2011. p. 4488–95. doi:10.1109/IROS.2011.6048620.

[2] Park YL, Chen BR, Pérez-Arancibia NO, Young D, Stirling L, Wood RJ, et al. Design and control of a bio-inspired soft wearable robotic device for ankle-foot rehabilitation. Bioinspir Biomim. 2014;9:17. doi:10.1088/1748-3182/9/1/016007.

Actuators

Type	Pneumatic actuator – McKibben artificial muscle
Location	On-board
Power	N/A
Maximum torque	N/A
Maximum speed	N/A
Bandwidth	2.40 Hz
Weight	N/A
Modular actuators	✗
Actuator specific characteristics	
Pnumatic muscles/leg	
Artificial muscle diameter/length	N/A / N/A
Artificial muscle maximum pressure	N/A
Artificial muscle stiffness	N/A
Actuator stiffness	N/A

Structure & interface attachment components

Allowable wearer maximum weight	N/A
Allowable wearer height range	N/A
Anthropomorphic considerations	Exoskeleton orthoses were custom molded to each subject
Configurable dimensions	N/A
Structure typology	Rigid structure
Braces/segment	1

Applicability & general characteristics

Target user	Adults
Application	Assistance
Unilateral/bilateral	Unilateral
Set up	Ambulatory
Exoskeleton weight	N/A
Total DoFs	2 (Knee_FE, Ankle_DP)
Actuated DoFs	2 (Knee_FE, Ankle_DP)
Walking speed	1.25 m/s

Control

Control strategies	Proportional myoelectric control

Electric power supply

Battery autonomy	N/A

Air source

Location	Off-board
Autonomy	N/A
Maximum pressure	N/A

Related publications

[1] Sawicki GS, Ferris DP. A pneumatically powered knee-ankle-foot orthosis (KAFO) with myoelectric activation and inhibition. J Neuroeng Rehabil. 2009;6:1–16. doi:10.1186/1743-0003-6-23.

Actuators

Type	Pneumatic actuator – PPAM
Location	On-board
Power	N/A
Maximum torque	N/A
Maximum speed	N/A
Badwidth	6 Hz-50 Nm; 20 Hz-10 Nm,
Weight	0.15 kg
Modular actuators	✘
Actuator specific characteristics	
Pnumatic muscles/leg	2
Artificial muscle diameter/length	N/A / N/A
Artificial muscle maximum pressure	N/A
Artificial muscle stiffness	N/A
Actuator stiffness	N/A

Structure & interface attachment components

Allowable wearer maximum weight	90 kg
Allowable wearer height range	N/A
Anthropomorphic considerations	NASA's anthropometric data [*]
Configurable dimensions	Braces position and orientation
Structure typology	Rigid structure
Braces/segment	1

Applicability & general characteristics

Target user	Adults
Application	Rehabilitation (MS)
Unilateral/bilateral	Unilateral
Set up	Treadmill-based (supportive lateral arm)
Exoskeleton weight	4.50 kg
Total DoFs	1 (Knee_FE)
Actuated DoFs	1 (Knee_FE)
Walking speed	0.70 m/s

Control

Control strategies	· Torque control<br>· Position control

Electric power supply

Battery autonomy	N/A

Air source

Location	Off-board
Autonomy	N/A
Maximum pressure	N/A

Related publications

[1] Beyl P, van Damme M, van Ham R, Vanderborght B, Lefeber D. Design and control of a lower limb exoskeleton for robot-assisted gait training. Appl Bionics Biomech. 2009;6:229–43. doi:10.1080/11762320902784393.

[2] Beyl P, Van Damme M, Van Ham R, Vanderborght B, Lefeber D. Pleated pneumatic artificial muscle-based actuator system as a torque source for compliant lower limb exoskeletons. IEEE/ASME Trans Mechatronics. 2014;19:1046–56. doi:10.1109/TMECH.2013.2268942.

[3] Beyl P, Knaepen K, Duerinck S, Van Damme M, Vanderborght B, Meeusen R, et al. Safe and Compliant Guidance by a Powered Knee Exoskeleton for Robot-Assisted Rehabilitation of Gait. Adv Robot. 2011;25:513–35. doi:10.1163/016918611X558225.

[*] NASA, 1978. Anthropometric source book, Vol. I: anthropometry for designers. NASA Scientific and Technical Information Office Hampton, VA, USA

Actuators

Type	Pneumatic actuator – McKibben artificial muscle	
Location	On-board	
Power	N/A	
Maximum torque	N/A	
Maximum speed	N/A	
Bandwidth	2.40 Hz	
Weight	N/A	
Modular actuators	✗	
Actuator specific characteristics		
Pnumatic muscles/leg		2
Artificial muscle diameter/length		N/A / 460 mm
Artificial muscle maximum pressure		N/A
Artificial muscle stiffness		N/A
Actuator stiffness		N/A

Structure & interface attachment components

Allowable wearer maximum weight	N/A
Allowable wearer height range	N/A
Anthropomorphic considerations	N/A
Configurable dimensions	N/A
Structure typology	Rigid structure
Braces/segment	1

Applicability & general characteristics

Target user	Adults
Application	Rehabilitation
Unilateral/bilateral	Unilateral
Set up	Ambulatory
Exoskeleton weight	1.21 kg
Total DoFs	1 (Ankle_DP)
Actuated DoFs	1 (Ankle_DP)
Walking speed	1.25 m/s

Control

Control strategies	Porportional myoelectric control

Electric power supply

Battery autonomy	N/A

Air source

Location	Off-board
Autonomy	N/A
Maximum pressure	N/A

Related publications

[1] Ferris DP, Gordon KE, Sawicki GS, Peethambaran A. An improved powered ankle-foot orthosis using proportional myoelectric control. Gait Posture. 2006;23:425–8. doi:10.1016/j.gaitpost.2005.05.004.

[2] Gordon KE, Sawicki GS, Ferris DP. Mechanical performance of artificial pneumatic muscles to power an ankle–foot orthosis. J Biomech. 2006;39:1832–41. doi:10.1016/j.jbiomech.2005.05.018.
[3]Sawicki GS, Ferris DP. Mechanics and energetics of level walking with powered ankle exoskeletons. J Exp Bilogy. 2008;211:1402–13. doi:10.1136/rmdopen-2015-000202.

Actuators

Type	Rigid actuator
Location	On-board/off-board
Power	N/A
Maximum torque	N/A
Maximum speed	1.50 m/s
Bandwidth	N/A
Weight	2.63 kg
Modular actuators	✗
Actuator specific characteristics	
Extra spring/spring stiffness value	✗

Structure & interface attachment components

Allowable wearer maximum weight	N/A
Allowable wearer height range	N/A
Anthropomorphic considerations	Participant measures for frame fitting
Configurable dimensions	N/A
Structure typology	Non-rigid frame (soft exoskeleton)
Structure weight	0.90 kg
Braces/segment	1

Applicability & general characteristics

Target user	Adults
Application	Assistance (Stroke)
Unilateral/bilateral	Unilateral
Set up	Ambulatory/treadmill-based (safety harness)
Exoskeleton weight	4.09 kg
Total DoFs	1 (Ankle_DP)
Actuated DoFs	1 (Ankle_DP)
Walking speed	1.25 m/s

Control

Control strategies	Position control

Electric power supply

Battery autonomy	N/A

Related publications

[1] Awad LN, Bae J, O'Donnell K, De Rossi SMM, Hendron K, Sloot LH, et al. A soft robotic exosuit improves walking in patients after stroke. Sci Transl Med. 2017;9. doi:10.1126/scitranslmed.aai9084.

[2] Bae J, De Rossi SMM, O'Donnell K, Hendron KL, Awad LN, Teles Dos Santos TR, et al. A soft exosuit for patients with stroke: Feasibility study with a mobile off-board actuation unit. In: 2015 IEEE International Conference on Rehabilitation Robotics (ICORR). Singapore (Singapore); 2015. p. 131–8. doi:10.1109/ICORR.2015.7281188.

Mooney et al. [1]

Actuators

Type	Rigid actuator
Location	On-board
Power	200 W (motor)
Maximum torque	N/A
Maximum speed	N/A
Bandwidth	N/A
Weight	0.749 kg
Modular actuators	✘
Actuator specific characteristics	
Extra spring/spring stiffness value	✘

Structure & interface attachment components

Allowable wearer maximum weight	N/A
Allowable wearer height range	N/A
Anthropomorphic considerations	Subjects with a foot size between 11 and 12.5
Configurable dimensions	N/A
Structure typology	Non-rigid structure (soft exoskeleton)
Structure weight	N/A
Braces/segment	2

Applicability & general characteristics

Target user	Adults
Application	Assistance
Unilateral/bilateral	Bilateral
Set up	Ambulatory
Exoskeleton weight	8.96 kg
Total DoFs	1 (Ankle_DP)
Actuated DoFs	1 (Ankle_P)
Walking speed	1.50 m/s

Control

Control strategies	· Torque control<br>· Zero torque control

Electric power supply

Battery autonomy	N/A

Related publications

[1] Mooney LM, Rouse EJ, Herr HM. Autonomous exoskeleton reduces metabolic cost of human walking during load carriage. J Neuroeng Rehabil. 2014;11:80. doi:10.1186/1743-0003-11-80.
[2] Mooney LM, Herr HM. Biomechanical walking mechanisms underlying the metabolic reduction caused by an autonomous exoskeleton. J Neuroeng Rehabil. 2016;13:4. doi:10.1186/s12984-016-0111-3.
